# Epigenetic regulation and T-cell responses in endometriosis – something other than autoimmunity

**DOI:** 10.3389/fimmu.2022.943839

**Published:** 2022-07-22

**Authors:** Dariusz Szukiewicz

**Affiliations:** Department of Biophysics, Physiology and Pathophysiology, Faculty of Health Sciences, Medical University of Warsaw, Warsaw, Poland

**Keywords:** epigenetic mechanisms, T cells, endometriosis, autoimmunity, T-cell reprogramming

## Abstract

Endometriosis is defined as the presence of endometrial-like glands and stroma located outside the uterine cavity. This common, estrogen dependent, inflammatory condition affects up to 15% of reproductive-aged women and is a well-recognized cause of chronic pelvic pain and infertility. Despite the still unknown etiology of endometriosis, much evidence suggests the participation of epigenetic mechanisms in the disease etiopathogenesis. The main rationale is based on the fact that heritable phenotype changes that do not involve alterations in the DNA sequence are common triggers for hormonal, immunological, and inflammatory disorders, which play a key role in the formation of endometriotic foci. Epigenetic mechanisms regulating T-cell responses, including DNA methylation and posttranslational histone modifications, deserve attention because tissue-resident T lymphocytes work in concert with organ structural cells to generate appropriate immune responses and are functionally shaped by organ-specific environmental conditions. Thus, a failure to precisely regulate immune cell transcription may result in compromised immunological integrity of the organ with an increased risk of inflammatory disorders. The coexistence of endometriosis and autoimmunity is a well-known occurrence. Recent research results indicate regulatory T-cell (Treg) alterations in endometriosis, and an increased number of highly active Tregs and macrophages have been found in peritoneal fluid from women with endometriosis. Elimination of the regulatory function of T cells and an imbalance between T helper cells of the Th1 and Th2 types have been reported in the endometria of women with endometriosis-associated infertility. This review aims to present the state of the art in recognition epigenetic reprogramming of T cells as the key factor in the pathophysiology of endometriosis in the context of T-cell-related autoimmunity. The new potential therapeutic approaches based on epigenetic modulation and/or adoptive transfer of T cells will also be outlined.

## 1 Introduction

**Figure 1:**
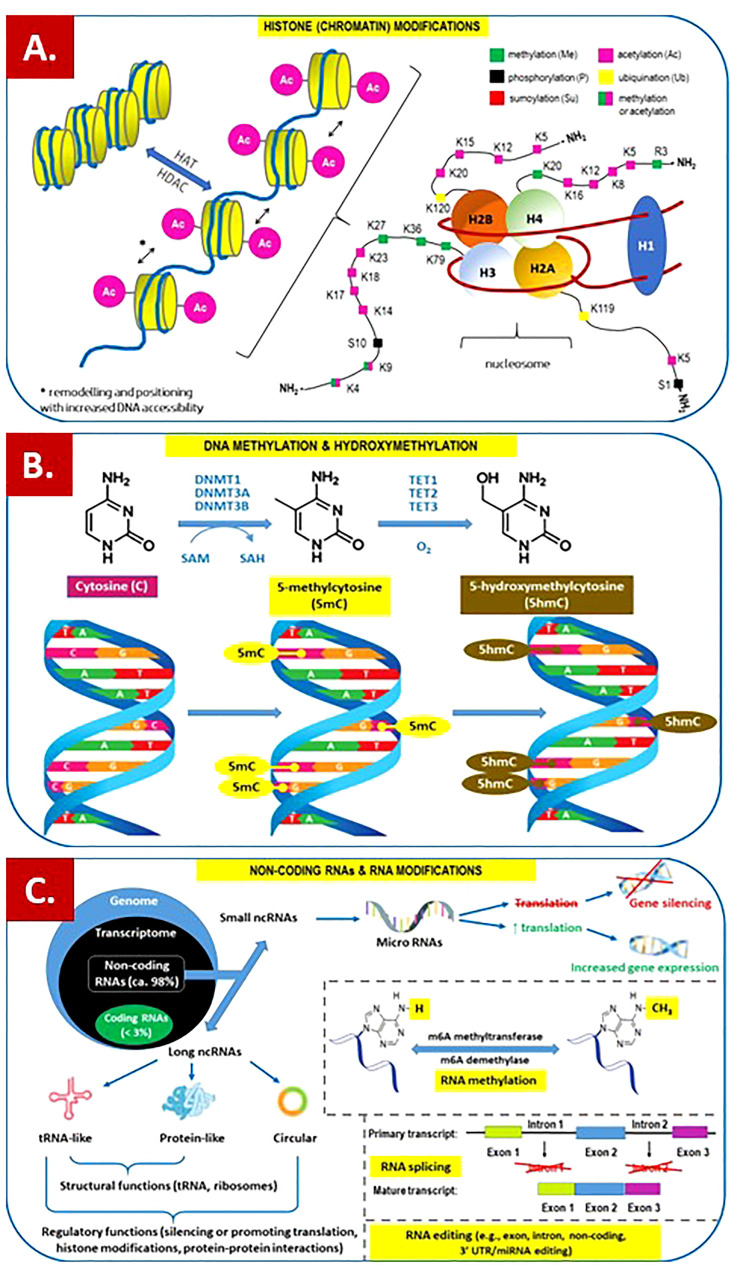
Overview of the main epigenetic mechanisms that regulate gene expression and may establish potentially heritable changes in gene expression without altering the underlying DNA nucleotide sequence. **(A)** Histone (chromatin) modifications. *On the left.* Chromatin remodeling is the dynamic modification of chromatin architecture to allow access of condensed genomic DNA to the regulatory transcription machinery proteins, and thereby control gene expression. For example, histone acetylation by HAT (histone acetyl transferase) increases DNA (chromatin) accessibility because acetylated histones cannot pack as well together as deacetylated histones. HDAC – histone deacetylase; *On the right.* Each nucleosome consists of two subunits, both made of histones H2A, H2B, H3 and H4, also known as core histones, with the linker histone H1 acting as a stabilizer. Histone post-translational modifications are covalent modifications of histones by phosphorylation on serine or threonine residues, methylation on lysine or arginine, acetylation and deacetylation of lysines, ubiquitylation of lysines and sumoylation of lysines. Histone modifications affect chromosome structure and function, especially during transcription and chromatin remodelling processes. **(B)** DNA methylation and hydroxymethylation. DNA can be modified at cytosine and adenine residues by the addition of chemical groups. Cytosines can be modified by methylation (5mc) or hydroxymethylation (5hmC), while adenines are modified by methylation. CpG islands (regions of the genome that contain a large number of CpG dunucleotide repeats) are DNA methylations regions in promoters known to regulate gene expression through transcriptional silencing of the corresponding gene. DNA methylation at CpG islands is crucial for gene expression and tissue-specific processes. DNMT – DNA methyltransferase; SAM – S-adenosylmethionine; SAH – S-adenosylhomocysteine; TET – ten-eleven-translocation (methylcytosine dioxygenase). (C) Non-coding RNAs (ncRNAs) and RNA modifications. - ncRNAs play an important role in transcription regulation by epigenetic machinery. Within RNA-induced silencing complexes (RISCs), miRNAs mediate the recognition and binding of RNAs that become targeted for degradation. lncRNAs are associated with other complexes and can activate or repress transcription. - RNA methylation is a post-transcriptional level of regulation. At present, more than 150 kinds of RNA modifications have been identified. They are widely distributed in messenger RNA (mRNA), transfer RNA (tRNA), ribosomal RNA (rRNA), noncoding small RNA (sncRNA) and long-chain non-coding RNA (lncRNA). - Alternative splicing (AS) of pre-mRNAs serves as an additional regulatory process for gene expression after transcription, and it generates distinct mRNA species, and even noncoding RNAs (ncRNAs), from one primary transcript. AS contributes to the diversity of proteins in eukaryotes as cells respond to signals from the environment. AS may lead to generation of ncRNAs, especially long noncoding RNAs (lncRNAs). RNA modifications, such as the RNA N6-methyladenosine (m6A) modification, have been found to regulate AS. **- RNA editing** is an important mechanism of genetic regulation that amplifies genetic plasticity by allowing the production of alternative protein products from a single gene. RNA editing involves the post-transcriptional insertion and deletion of nucleotides (e.g., uridylate – UMP) within nascent transcripts. RNA editing has been observed in mRNAs, tRNAs, and rRNAs, in mitochondrial and chloroplast encoded RNAs, as well as in nuclear encoded RNAs.

Epigenetics is focused on studying changes in gene expression, including mitotically and/or meiotically heritable phenotype modifications that arise from changes in chromosomes but do not involve alterations in the DNA sequence (1). Among the better-known epigenetic mechanisms of histone protein posttranslational modifications, higher-order chromatin reorganization, DNA methylation and hydroxymethylation, nucleosome remodeling/repositioning, RNA editing, and noncoding RNA regulation should be mentioned (2, 3) (**Figure 1**). A variety of these mechanisms to the differentiation of environmental stimuli that trigger the specified epigenetic modification (4). Changes in gene expression without modification of DNA sequence can be induced by several factors, including age, sex, diet, smoking, and other stimulants, exposure to viruses and bacteria, stress, disease state, and chronic alcohol abuse (5, 6). For example, chronic exposure to ethanol modifies DNA and histone methylation, histone acetylation, and microRNA expression (7). Undoubtedly, epigenetic mechanisms play a key role in ensuring homeostasis or maintenance of a constant internal environment. On the other hand, abnormal epigenetic regulation of the human body systems, including the immune system, may predispose to certain diseases or contribute to the development of both rare syndromes and diseases of high prevalence in human populations (8, 9). All epigenetic changes are reversible. This may explain the fact that these modifications are rarely maintained in future generations in humans, even if they have been repeated in numerous cell cycles (10). Pathoepigenetics is an emerging new field dealing with the description of pathologic changes elicited by epigenetic defective reprogramming (11). Considering the reversibility of these changes, elucidation of the epigenetic consequences of environmental-host interactions may have important therapeutic implications (12).

In autoimmune diseases, the body’s immune system mistakenly identifies its own healthy tissues as foreign and attacks them. There are over 100 different autoimmune diseases, and the symptoms and effects vary from case to case (13, 14). Most of these diseases run with a more or less evident inflammatory response that can affect many parts of the body. It is estimated that autoimmune diseases affect 3-5% of the population, and widespread diseases include systemic lupus erythematosus (SLE), Hashimoto’s autoimmune thyroiditis, diabetes mellitus type 1, rheumatoid arthritis (RA), Graves’ disease, vitiligo, and celiac disease (CD) (15). There is an upward trend in the prevalence of autoimmunity in developed countries, as was recently found by analysis of the prevalence of the most common biomarker of immunization, antinuclear antibodies (ANA), in the US population (16).

The immune effectors involved in the autoimmune response and related disorders include cells such as natural killer cells (NKs), cytotoxic lymphocytes (CTLs), macrophages, mast cells, and molecules such as antibodies, the complement system, and cytokines (including chemokines). The role of T cells in the pathological immune response, resulting in self-injury, still arouses considerable interest (17). T cells are T lymphocytes, one of the important white blood cells of the immune system. Having the T-cell receptor (TCR) on its surface, a protein complex that is responsible for recognizing fragments of antigens as peptides bound to major histocompatibility complex (MHC) molecules, T cells play a central role in the adaptive immune response. The pathogenesis of autoimmune diseases encompasses both T-cell-induced abnormal (cytotoxic) activation of lymphocytes and macrophages and T-cell-mediated maturation of B cells (B lymphocytes) into memory B cells and plasma cells that produce antibody molecules closely modeled after the receptors of the precursor B cell. The above functions are provided by the T-cell subpopulation named T helper cells (Th cells), also known as CD4+ cells (CD4-positive cells), as they express the CD4 glycoprotein on their surface (17, 18). On the other hand, a T-cell subpopulation named regulatory T cells (Tregs) is essential to maintain immune homeostasis and prevent autoimmune disorders (19, 20). It has been proven that the entire population of T cells is subjected to epigenetic mechanisms that largely govern its maturation and differentiation (21, 22). Thus, precise identification of functional epigenetic pathways in T-cell maturation/differentiation and the modifiers responsible for dysregulation of these processes may provide further insight into the nature of autoimmunity in the context of therapeutic methods.

Endometriosis is a common gynecological disorder affecting approximately 10% (range of 5 to 15%) of reproductive-aged women, whereas significantly higher percentages of endometriosis-related treatments (25 to 50%) have been administered among infertile female patients (23, 24). The term “endometriosis” refers to a condition in which endometrial tissue appears outside the uterine cavity (25). Such endometrial foci may be located either endopelvically or extrapelvically. Abnormally implanted endometrial tissue is typically found in the pelvis, including ovaries, ovarian fossa, fallopian tubes, uterine wall (endometriosis interna or adenomyosis), broad ligaments, round ligaments, uterosacral ligaments, appendix, large bowel, ureters, bladder, or rectovaginal septum (26, 27). Extrapelvic locations of endometriosis are rare. The ectopic endometrium is biologically the same as basal intrauterine endometrial tissue and – because endometriosis cells express estrogen receptors (ERα, Erβ, and GPER) and P4 receptors (PR-A and PR-B) – grows and undergoes cyclic proliferation and breakdown like the ectopic endometrium (28, 29). The local inflammatory response within the endometrial foci is accompanied by pain, including significantly compromising quality of life dyspareunia and dysmenorrhea as well as more serious complications related to fibrosis, scar tissue formation, and adhesions during repair processes (25, 30). A vicious circle of disease arises, where it is difficult to determine whether the inflammatory process favors the development of endometriosis foci or whether the endometriosis foci induce the inflammatory process (31, 32). As a result, patients with endometriosis are less likely to become pregnant and have a successful pregnancy outcome (33). Moreover, it has also been reported that women with endometriosis have a higher incidence of cancer and autoimmune diseases (34, 35). Considering the latter, it was reported that women with endometriosis are at greater risk for autoimmune diseases such as RA, multiple sclerosis (MS), SLE, Sjögren’s Syndrome (SS), and inflammatory bowel disease (IBD) (36). Endometriosis shares several similarities with these autoimmune diseases, including elevated levels of cytokines, decreased cell apoptosis, and T- and B-cell abnormalities.

The immune system is responsible for eliminating cells that are in ectopic sites, and the failure of this elimination in endometriosis is due either to resistance of endometriotic cells to be eliminated by immune cells or to a deficit in the immune response (35, 37). The coexistence of endometriosis and autoimmunity is a well-known occurrence. However, endometriosis has not yet been classified as an autoimmune condition. Despite the still unknown etiology of endometriosis, much evidence suggests the participation of epigenetic mechanisms in the etiopathogenesis of the disease, including immune dysfunction (38, 39). The main rationale is based on the fact that heritable phenotype changes that do not involve alterations in the DNA sequence are common triggers for hormonal, immunological, and inflammatory disorders, which play a key role in the formation of endometriotic foci (39).

This review aims to present the state of the art in recognition epigenetic reprogramming of T cells as the key factor in the pathophysiology of endometriosis. The new potential therapeutic approaches based on epigenetic modulation and/or adoptive transfer of T cells will also be outlined.

## 2 T cells and immune response

The hallmark of the adaptive immune system is clonal expansion of lymphocytes, including a rapid increase in T cells from one or a few cells to millions. T cells play a crucial role in the regulation of the immune system, providing a highly specific, long-lasting and – considering T memory cells – long-term defense mechanism against nonself-pathogens (40). Autoantigens are the result of mutation, neoantigen formation, or exposure of previously hidden self-antigens (41). Immunologic tolerance or a state of unresponsiveness in which lymphocytes remain alive but cannot exert effector functions against a particular antigen ensures a lack of reactivity to self-antigens (autoantigens) (42). In the process of central tolerance, self-reactive T cells possessing receptors specific for autoantigens are eliminated *via* apoptosis at an early stage in lymphoid cell development. Some CD4+ T cells receive signals in the thymus that select them to differentiate into “natural” T regulatory cells (nTregs), which express the FoxP3 transcription factor and suppress the immune response by both direct and indirect mechanisms. Peripheral tolerance ensures that self-reactive T cells from peripheral tissues are deleted (apoptosis), become anergic (functionally unresponsive to antigen), or can differentiate into “induced” Tregs (iTregs, formerly known as suppressor T cells) (43). Thus, the above two major subsets of CD4+ CD25+ Foxp3+ Tregs (nTregs and iTregs) are essential to the balance between pro- and anti-inflammatory responses. Even temporary malfunction of these checkpoints may cause uncontrolled expansion of these defective (self-reactive) T cells with subsequent development of autoimmunity (42, 43). Aiming to learn about the etiopathogenesis, still accumulating knowledge about the pathways and mediators of the T-cell-dependent autoimmune response includes the T-cell receptor (TCR), T-cell-related cytokines, and defective genes responsible for T-cell regulation and function (42–44). TCR signaling at the level of the membrane of T cells plays a key role in regulating T-cell homeostasis, activation, expansion, and effector function upon recognition of cognate foreign or self-antigens. The specificity of action and properties of the TCR repertoire are acquired during selection, a process in the thymus gland (45, 46). The binding between TCRs and antigens, including autoantigens, is of relatively low affinity and may gradually disengage, which leads to situations in which many TCRs recognize the same antigen peptide and many antigen peptides are recognized by the same TCR (47). TCR forms a TCR complex with six chains of cluster of differentiation 3 (CD3), kinases, coreceptors, and ligands (48).

### 2.1 Epigenetic mechanisms influencing TCR signaling and autoimmunity

**Figure 2:**
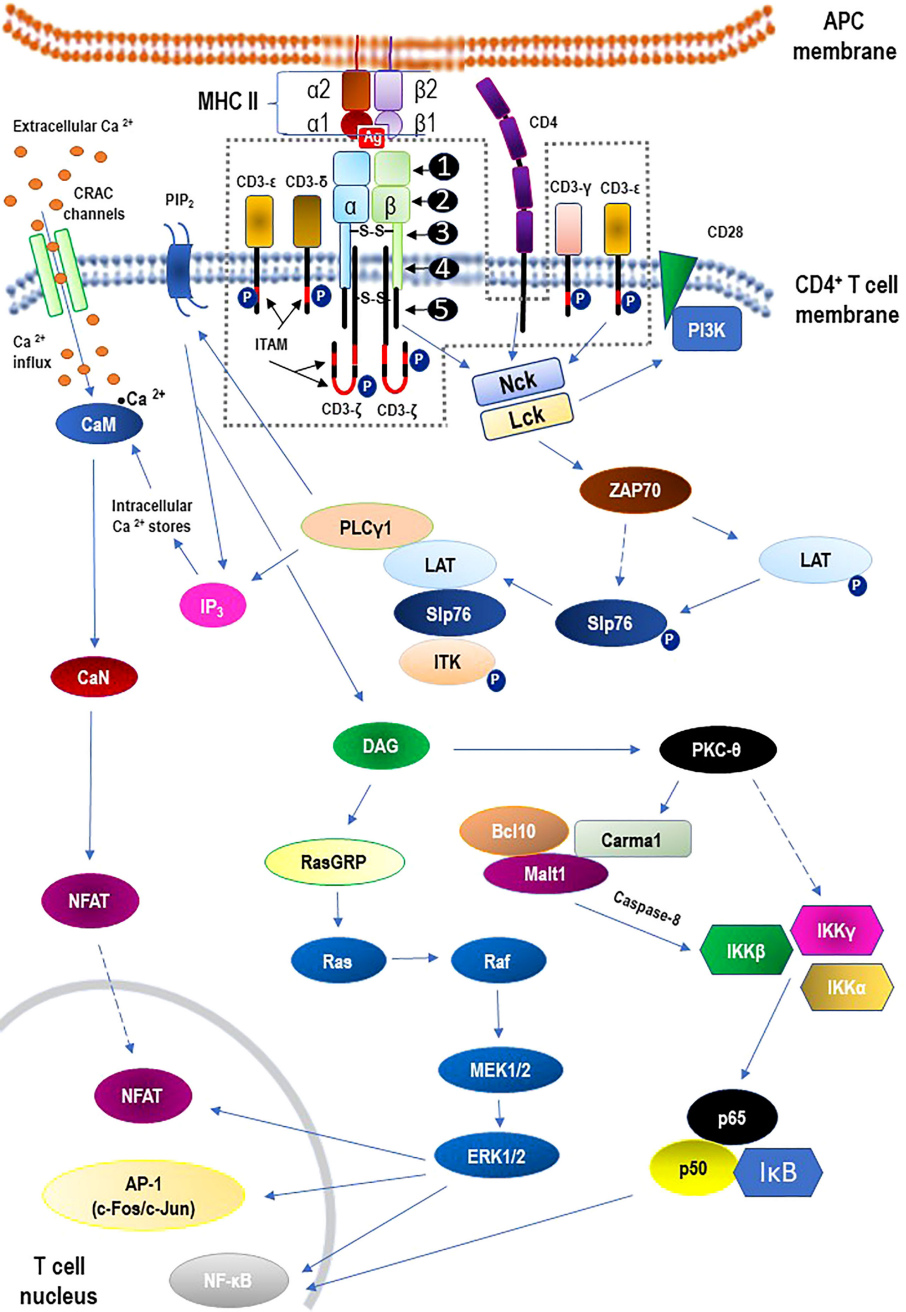
Structure of the αβ T cell receptor (TCR) and the TCR-CD3 complex (the area within the dashed line) including main signaling pathways. - TCR structure: ❶ - variable region; ❷ - constant region; ❸ - hinge region; ❹ - transmembrane region; ❺ - cytoplasmatic tail. - The core TCR signaling complex consists of two TCR chains (αβ heterodimer) that are noncovalently coupled to three dimeric signaling molecules named the cluster of differentiation 3 (CD3): CD3ϵδ, CD3ϵγ, and CD3ζζ. - Signaling *via* the TCR/CD3 antigen receptor complex is activated after interaction of the TCR with cognate peptide antigen bound to a major histocompatibility complex (MHC) molecule on the surface of antigen-presenting cells (APC), and co-stimulation by co-receptor molecules such as CD28. An early event in the proximal signaling of TCR is the involvement and activation of a set of protein tyrosine kinases (PTKs), such as LCK, FYN, and ZAP-70, that are important components required for activation of TCR signaling through tyrosine phosphorylation on CD3. The proximal TCR signaling is followed by the activation of multiple distal signaling cascades, such as: *• Ca^2+^–calmodulin (CaM) – calcineurin (CaN) – nuclear factor of activated T-cells (NFAT); • diacylglycerol (DAG) – Ras guanyl nucleotide releasing protein (RasGRP) – Ras – proto-oncogene serine/threonine-protein kinase (Raf) – dual-specificity tyrosine/threonine protein kinases (MEK1/2) – extracellular signal-regulated kinases 1/2 (ERK1/2);* • *protein kinase C-theta (PKCθ) – I kappa B kinases (IKKα, IKKβ, IKKγ) – nuclear factor kappa B (NF-κB).* These signaling cascades, regulated largely by epigenetic mechanisms, finally bring out the diverse phenotypic effects, as they control many aspects of T cell biology. For the sake of clarity of the diagram, the presentation of the negative regulation (downregulation) of TCR-mediated signaling has been abandoned. See the main text (2.2. TCR signaling) for details. α1, α2, β1, β2 – domains α1 and α2 and β1 and β2 of the chains (α and β, respectively) that form heterodimeric MHC-II complex; Ag – antigen; AP-1 – activator protein 1; Bcl10 – B cell lymphoma 10; Ca^2+^ – calcium; CaM – calmodulin; CaN – calcineurin; Carma1 – caspase recruitment domain membrane-associated guanylate kinase protein 1; c-Fos/c-Jun –AP-1-forming dimer of proto-oncogenes; CRAC – calcium release-activated Ca^2+^; DAG – diacylglycerol; IκB – kinase (IKK) complex containing IKKα, IKKβ, and IKKγ; IKKα – I kappa B kinase α; IKKβ – I kappa B kinase β; IKKγ – I kappa B kinase γ; IP_3_ – inositol trisphosphate; ITAM –immunoreceptor tyrosine-based activation motif; ITK – interleukin-2 inducible tyrosine kinase; LAT – linker activation of T cells; Lck – leukocyte-specific tyrosine kinase; Malt 1 – mucosa-associated lymphoid tissue protein 1; Nck – adaptor protein non-catalytic region of tyrosine kinase (Nck); NFAT – nuclear factors of activated T cells; NF-κB – nuclear factor kappa B; P –phosphorylated proteins; p50 – regulatory subunit of the NF-kB complex; p65 – subunit of NF-κB; PI3K – phosphatidylinositol-3 kinase; PIP2 – phosphatidylinositol bisphosphate; PKC-theta – protein kinase C-theta; PLCγ1 – phospholipase C gamma 1; RasGRP – Ras guanyl nucleotide releasing protein; Slp76 – SH2-domain containing leukocyte protein of 76 kDa; ZAP70 – zeta-activated protein 70 kDa.

The TCR is a member of the immunoglobulin superfamily, a large protein superfamily of cell surface and soluble proteins that are involved in the recognition, binding, or adhesion processes of cells. This means that the molecules of TCR share structural features with immunoglobulins (antibodies) (49). It is beyond the scope of this review paper to discuss the structure of the TCR or the TCR-CD3 complex. However, for a quick overview of these issues, please refer to **Figure 2**. with descriptive legend that includes key components of the TCR signaling pathway.

Based on numerous data to date, it can be assumed that TCR signaling is inherently involved in the control of adaptive immune responses but also in the acquisition of immunocompetence by T cells and their development and differentiation (50). As these processes are difficult to separate clearly, they will be discussed together in the context of epigenetic mechanisms, including those leading to autoimmunization. It is worth noting that, as long as histone protein posttranslational modifications, higher order chromatin reorganization, DNA methylation, hydroxymethylation or acetylation, and various noncoding RNA-mediated processes are thought to influence gene expression mainly at the level of transcription, other steps in the process (e.g., translation) may also be regulated epigenetically (51).

During early lymphocyte development, Ig and TCR variable region genes are assembled from germline component variable (V), diversity (D), and joining (J) gene segments (52). Such V(D)J recombination at antigen receptor (AR)-encoding loci (Tcra, Tcrb, Tcrd, and Tcrg) expressed in T cells is initiated by recombination activating proteins 1 (RAG1) and RAG2 *via* the introduction of DNA double strand breaks (DSBs) between the V, D, and J coding segments and flanking recombination signal (RS) sequences (53). In αβT-cells, this leads to the subsequent expression of TCR β and α chains (54). Such T-cell receptor gene assembly by V(D)J recombination proceeds *via* successive Dβ-to-Jβ and Vβ-to-DJβ as well as Dα-to-Jα and Vα-to-DJα rearrangements. Basically, V(D)J recombination is strictly controlled at various levels, including these checkpoints that rely on modulation of gene accessibility to the recombination machinery. Biochemical changes in chromatin arrangement and structural modifications of chromosomal organization and positioning form the epigenetic basis for functional development of the TCR antigen (55). Research on the concept of the accessibility model assuming that locus-specific control and temporal ordering of V(D)J recombination primarily involve the modulation of locus and/or gene segment accessibility to a common VDJ recombinase led to groundbreaking findings (56, 57). It was established that both the lineage specificity and temporal ordering of gene rearrangement are reflected in *in vitro* recombinase cleavage of RSs flanking Ig and TCR gene segments within chromatin in isolated nuclei (58, 59). This means that unlike “compact” chromatin within recombination-inert regions, chromatin at gene segments/loci undergoing V(D)J rearrangement fulfils the criteria of an “open” (noncompacted) configuration (60, 61). Referring to gene expression, the “compact” and “open” regions of chromatin are regions of silent and active transcription, respectively (62). When analyzing, for example, the Tcrb locus, a lightly packed form of chromatin, euchromatin, is linked both locally and in a stage-specific way with Dβ-to-Jβ recombination events taking place with the assistance of germline transcription (GT), accessibility to restriction enzymes, enrichment in histone H3/H4 acetylation and H3K4 methylation, lack of CpG methylation, and diminished nucleosome abundance (54). However, from the double-negative 1-4 to the double positive (DN1-4-to-DP) thymocyte transition onward, lack of GT and decreased histone H3/H4 acetyl lysine (H3/H4ac) predominated along chromosomal regions comprising non-rearranged 5′Vβ genes (54). Expression of a productively rearranged VβDJβ CJ (hereafter VDJ+) and formation of a primary receptor, namely, the pre-TCR, triggers further differentiation into DN4 cells and subsequently CD4/CD8 DP cells. This developmental shift is known as β-selection because it selects for cells that have successfully rearranged their TCR-β chain locus. V(D)J recombination is arrested during this period of cell differentiation. Finally, it resumes in DP cells by selectively targeting the TCRα locus to achieve Vα-to-Jα joining, followed by further selection events involving the completed αβTCR (63). In the studies that involved insertion of a Dβ–Jβ recombination substrate into the endogenous Vβ14 gene segment, accessibility for recombination upon the inserted reporter remained dependent on epigenetically modulated chromatin conformation (64). The significance of this euchromatin-to-heterochromatin transition in health and disease is the subject of intense research aimed at identification of the combination of histone marks (e.g., H3K4ac, H3K4me) that possibly produce restriction at V(D)J rearranging loci (62, 63, 65). It is possible that active epigenetic marks are established through the recombining gene segments and associated RSs before AR V(D)J assembly by interaction with either sense or antisense GT (66, 67). Binding of RAG1 and RAG2 proteins that occurs in a highly focal manner to a small region of active chromatin with high levels of H3K4ac and H3K4me may suggest a close link between RNA polymerase (Pol) II-mediated transcription and epigenetic tagging at these sites of the Tcrb locus, precisely encompassing Tcrb J and proximal D gene segments, in a developmental stage- and lineage-specific manner (68).

Euchromatin and related epigenetic modification to the DNA packaging protein histone H3 at V(D)J rearranging loci exerts effects not limited to the gene accessibility only, but it also has a significant and direct impact on the chromosomal environment in the context of V(D)J recombinase tethering and enhancement of catalytic activity. It was demonstrated that the plant homeodomain (PHD) zinc finger of RAG2 binds much more strongly to histone H3 trimethylated at K4 (H3K4me3) (69–71). In addition, H3K4me3 plays a significant role in the stimulation of purified RAG enzymatic activity at both the nicking (2- to 5-fold) and hairpinning (3- to 11-fold) steps of V(D)J recombination (72). Similarly, the N-terminal part of RAG1 contains a short for Really Interesting New Gene (RING) finger domain preferentially interacting directly with and promoting monoubiquitylation of histone H3. Monoubiquitination of histone H3 (H3ub1) could play a role in regulating the joining phase of chromosomal V(D)J recombination (73). It was established that RAG1 binds specifically to AR gene segments in a cell-type and stage-specific manner, whereas RAG2 has a much broader chromosome binding spectrum because it interacts with H3K4me3-enriched regions genome-wide (68).

Interacting with forkhead Box P3 (Foxp3) transcription factor complexes, TCR signaling plays central roles in Treg differentiation, maintenance, and functional maturation (74). During differentiation, Tregs recognize their cognate antigens and receives TCR signals before initiation of Foxp3 transcription, which is triggered by TCR-induced transcription factors, including nuclear factor of activated T cells (NFAT), activator protein 1 (AP-1), and nuclear factor kappa-light-chain-enhancer of activated B cells (NF-κB) (75). Naturally, derived Tregs are characterized by stable expression of the transcription factor Foxp3 and characteristic epigenetic imprinting at the Foxp3 gene locus. Foxp3 seizes TCR signal-induced transcriptional and epigenetic mechanisms by interacting with AML1/Runx1 and NFAT. Thus, Foxp3 modifies the gene expression dynamics of TCR-induced genes, which constitute cardinal mechanisms for Treg-mediated immune suppression and related self-tolerance and prevention of autoimmunity (74). It is essential that the NF-κB signaling pathway acts as a versatile regulator of Foxp3 expression during normal T-cell development and enhancing the signal strength of the NF-κB pathway induces Foxp3 expression in T cells, including Tregs (75). Interestingly, recent studies have revealed exciting new roles for NF-κB related to its nontranscriptional activities. It has been proven that NF-κB can also activate diverse epigenetic mechanisms that mediate extensive chromatin remodeling of target genes to regulate T-cell activities. Even epigenetic effects on genes encoding different NF-κB subunits may modulate T-cell inflammatory responses (75–77).

T cells depend on mammalian target of rapamycin kinase (mTOR) signaling to sense and integrate immune signals from dendritic cells (including antigenic signals, costimulatory molecules, and cytokines), environmental cues derived from growth factors and immunoregulatory factors, and nutrients (78). Another manifestation of the epigenetic regulation of TCR signaling in T cells arises from the posttranscriptional modulation of mTOR complex components mTOR and Rictor mRNAs by the microRNAs Let-7 and MiR-16. These results for CD4+ T cells demonstrated that microRNAs regulate the expression of mTOR components in T cells and that this regulation is critical for adjustable mTOR activity. Hence, influencing the interpretation of TCR signaling, microRNAs contribute to the discrimination between T-cell activation and anergy (79). Another important mechanism of miRNA regulation of CD4+ Treg development *via* modulation of the genes within the mTOR signaling pathway is related to miR-15b/16, miR-24, and miR-29a (80). Suppression of mTOR signaling is essential for induction of iTregs from naïve CD4(+) T cells, and the mTOR complex 2 (TORC2) component, Rictor, contains a functional target site for miR-15b/16. It was confirmed that downregulation of Rictor produces a significant reduction in mTOR signaling as measured by phosphorylation of the downstream target, ribosomal protein S6. In line with the knowledge that CD4+ Tregs are essential for controlling immune responses and preventing autoimmunity, the overexpression of miR-15b/16 in conventional CD4+ T cells adoptively transferred into Rag2(-/-) mice increased the *in vivo* development of peripheral Tregs and diminished the severity of autoimmune colitis (80).

Signal transduction may also be regulated based on reciprocal allosteric regulation of TCR phosphorylation related to cholesterol and ligand binding to the TCRβ transmembrane region (81). It was reported that cholesterol bound to the TCRβ transmembrane region keeps the TCR in a resting, inactive conformation that cannot be phosphorylated by active kinases (82). This ensures that the αβ T-cell remains quiescent in the absence of antigenic peptide-MHC (the TCR’s ligand) at the variable regions of TCRαβ and decreases the sensitivity of the T-cell toward stimulation. Only TCRs that spontaneously detach from cholesterol can achieve the active conformation (named primed TCRs) that is prone to phosphorylation. On the other hand, cholesterol binding to TCRβ leads to an increased formation of TCR nanoclusters, increasing the avidity of the TCRs toward the antigen and thus increasing the sensitivity of the αβ T-cell (83). The latter mechanism seems to be relevant in autoimmunity, as evidence is building up that cholesterol accumulation in leukocytes is causally associated with the production of autoantibodies (84). In contrast to TCRαβ, TCRγδ does not bind to cholesterol and might be regulated in a different manner (83).

Accumulation of intracellular lipid (cholesterol-containing) droplets in CD4+ T cells, coexisting with elevation of serum triglycerides and cholesterol, was observed in many autoimmune diseases, including rheumatic arthritis, SLE, and psoriasis (85–87). Even if it remains speculative, many authors postulated that lowering blood lipids or normalizing the lipid profile may limit T-cell-dependent autoantibody responses (87–90). It is worth noting that epigenetic modulation of cholesterol binding into TCRs may trigger a specific functional state of TCRs, both the resting and the primed (83, 91–93).

Epigenetic influences on TCR signaling should also be analyzed in the context of counteracting affect, i.e., considering that TCR signaling affects epigenetic modulation (94). T-cell activation induces changes in DNA methylation and acetylation, creating broad and lasting genetic modifications (95, 96). Typical markers of altered access to gene transcription include histone H3 lysine 27 trimethylation (H3K27Me3) and histone H3 lysine 27 acetylation (H3K27Ac), but the repertoire of epigenetic activity also includes phosphorylation, nitrosylation, glycosylation, lipidation, ubiquitination, and (small ubiquitin-related modifier) SUMOylation (97). In the case of histones H3K27Me3 and H3K27Ac, methylation is associated with a closed chromatin conformation that prevents gene transcription, whereas acetylation correlates with an “open” (permissive for transcription) chromatin conformation. Analogously, histone methyltransferases and deacetylases are associated with the silencing of gene expression, and histone demethylases and acetyltransferases promote gene expression (94). In addition, difficult to predict in an individual case, the effects of inadequate methylation/demethylation and acetylation/deacetylation on the chromatin conformation should be considered (21, 98).

For example, the chromatin-modifying enzyme enhancer of zeste homolog 2 (EZH2), the functional unit of polycomb repressive complex 2 (PRC2), is a histone methylase that plays a key role in regulating various aspects of T-cell immunobiology, such as Foxp3+ Treg stability (99, 100). The immune homeostasis associated with normal Treg function requires the induction of EZH2 in response to costimulation with CD28, an extracellular cue intrinsically required for Treg maintenance. Treg-specific deprivation of EZH2 resulted in spontaneous autoimmunity with reduced Foxp3(+) cells in nonlymphoid tissues and impaired resolution of experimental autoimmune encephalomyelitis (100).

Dysregulation of the balance between subsets of CD4+ T cells, Tregs, and Th17 cells may be involved in the pathomechanism of several disorders, including autoimmune disease, cancer, and chronic inflammatory conditions. The Treg/Th17 balance depends on many factors involved in the differentiation of these cells, such as TCR signals, cytokines, and metabolic and epigenetic regulators. The latter or posttranslational modifications modulate the activity of forkhead Box P3 (Foxp3), retinoic acid-related orphan receptor gamma t (RORγt), and signal transducer and activator of transcription (STAT)s. Thus, insufficient posttranslational (epigenetic) modifications of Treg/Th17 differentiation and/or balance may lead to autoimmune diseases (97).

Epigenetic influence applies to all three steps of TCR signaling, i.e., signal reception, transduction, and the response triggered by the signal. Thus, aberrant chromatin landscapes following T-cell activation were demonstrated in various autoimmune diseases, including rheumatoid arthritis, SLE, Grave’s disease, and type 1 diabetes mellitus (T1D) (94, 101). Although TCR signaling defects are associated with mediating pancreatic β cell autoimmunity in T1D, the disease is often complicated with other autoimmune diseases, and anti-islet autoantibodies precede the clinical onset of disease (102). Typically, T1D co-occurs in most cases with other common organ-specific autoimmune diseases, such as autoimmune thyroiditis (predominantly), celiac disease, and gastritis (102, 103). Accordingly, Teffs isolated from nonobese diabetic (NOD) mice display a particular chromatin conformation that allows not only easier access to T1D-associated genetic loci but also access to the genes involved in other autoimmune disorders (104–106).

Expression of foxp3 in naïve T cells during induction of differentiation into induced Foxp3+ regulatory T cells (iTregs) occurs with participation of suboptimal (weaker than maximal) TCR stimulus or TCR stimulus in conjunction with TGF-β signaling. It was demonstrated that optimal (strong) activation of TCR in terms of both ligand affinity and duration results in specific enrichment at the foxp3 locus with the accumulating DNA (cytosine-5)-methyltransferase 1 (DNMT1) and DNMT3b. This in turn leads to increased CpG methylation and inhibits foxp3 transcription (107). Regardless of the transcription factor activation, TCR and TGF-β signals exert epigenetic effects on DNMT1 to modulate the expression of foxp3 by increasing CpG methylation. Augmentation of DNMT1 is regulated through at least two posttranscriptional mechanisms. The first assumes that a strong TCR signal inactivates constitutively active glycogen synthase kinase-3 beta GSK3β to rescue DNMT1 protein from proteasomal degradation. The second mechanism is based on evidence that a strong TCR signal suppresses miR-148a to derepress DNMT1 mRNA translation (107). The opposite effect is related to TGF-β signaling, which antagonizes DNMT1 accumulation *via* activation of the p38 mitogen-activated protein (p38 MAP) kinase pathway (107, 108). In addition, regulation of foxp3 transcription, which may be important for the induction of self-tolerance and the control of autoimmunity, depends on the production of NF-κB-dependent cytokines (e.g., TNFα, IFN-γ, IL-17 and IL-9) by the T cells themselves (109). In addition to its well-documented transcriptional activity, the NF-κB or NF-κB subunit proto-oncogene RelB (RelB) can also trigger diverse epigenetic mechanisms that mediate extensive chromatin remodeling and histone modifications of target genes to regulate T-cell fate decisions (76).

Vitamin C (l-ascorbic acid), a multifunctional water-soluble antioxidant substance, serves as an essential cofactor for many enzymes, including those influencing epigenetic modulation of gene expression (110, 111). In addition, vitamin C can significantly affect T-cell differentiation and may interfere with T-cell signaling (112). Ascorbic acid was discovered as a cofactor for ten-eleven translocation (TET) methylcytosine dioxygenases that use Fe(II) and 2-oxoglutarate as cosubstrates and are responsible for DNA demethylation. Vitamin C also serves as a likely cofactor for some Jumonji C (JmjC) domain-containing histone demethylases that catalyze histone demethylation (113). Thus, vitamin C deficiency can influence demethylation of both DNA and histones, further leading to different phenotypic presentations with an increased possibility of autoimmune disorders. DNA hypomethylation was demonstrated in T cells from patients with SLE, suggesting the development of autoimmunity by decreasing DNA methyltransferase expression, modifying DNA methylation patterns, and altering gene expression (114, 115). DNA methylation is also regulated in part by the extracellular signal-regulated kinase (ERK) pathway, which is influenced by vitamin C, and ERK pathway signaling is diminished in lupus T cells (116). Interestingly, a lack of vitamin C in scurvy may mimic SLE (117).

Finally, signals beside the TCR receptor may modulate the epigenetic landscape of T-cell subpopulations. For example, Treg epigenetic and functional identity is modulated by interleukin-2 (IL-2) in a TCR-independent manner by regulating the positioning of the pioneer factor special AT-rich sequence binding protein 1 (Satb1) in CD4+ thymocytes and subsequently controlling the genome-wide chromatin accessibility of thymic-derived Tregs (118). Thus, in addition to TCR triggering, the immunomodulatory action of IL-2 contributes to the selection of Foxp3+CD4+ Tregs, the functionally stable cell lineage indispensable for the maintenance of immunological self-tolerance and safeguarding immune homeostasis *in vivo* (118–120).

**Figure 3:**
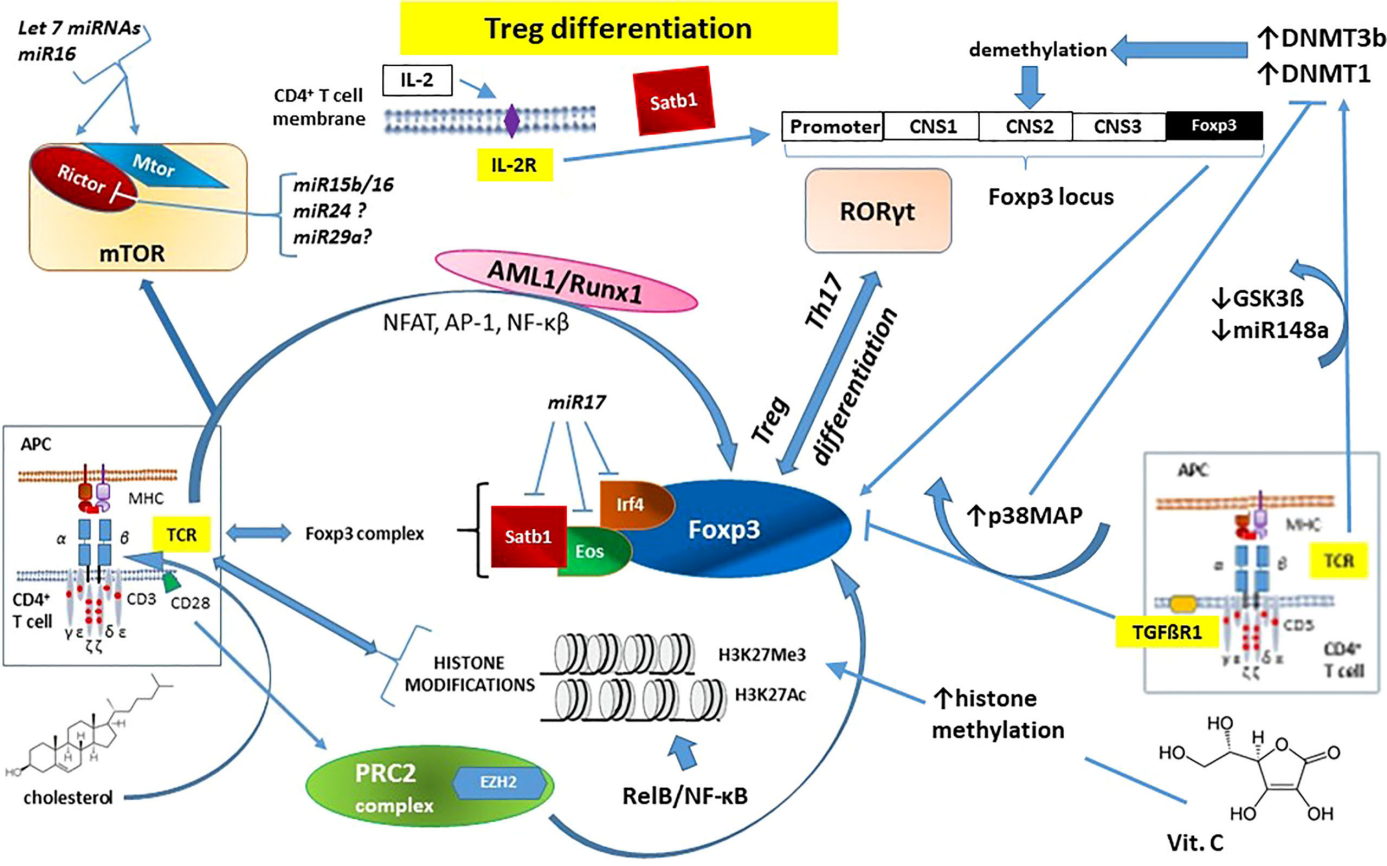
The essential role of epigenetic changes in regulatory T cell (Treg) development and function on the example of forkhead box P3 (Foxp3) **-** the master regulatory protein involved in Treg-mediated immune system responses. Noticeable that IL-2 action does not involve TCR signaling pathway. See the main text (Chapter 2.2.1. Epigenetic mechanisms influencing TCR signaling and autoimmunity) for details. AML1/Runx1 – Acute myeloid leukemia 1 protein or Runt-related transcription factor 1; AP-1 – activator protein 1; CD – cluster of differentiation (cell surface marker); CNS1-3 – conserved non-coding sequences; APC – antigen-presenting cells; DNMT – DNA methyl-transferase; Eos – transcription factor, member of the Ikaros Zinc Finger (IkZF) family of transcription factors; EZH2 – Enhancer of Zeste Homolog 2; Foxp3 – forkhead box P3 protein; GSK3β – glycogen synthase kinase-3 beta; H3K27Ac – acetylation of the lysine residue at N-terminal position 27 of the histone H3; H3K27Me3 – tri-methylation of lysine residue at N-terminal position 27 on the histone H3; IL-2 – interleukin 2; IL-2R – interleukin 2 receptor; Irf4 – Interferon regulatory factor 4; miRNA/miRNAs –microRNA/microRNAs; mTOR – mammalian target of rapamycin kinase; mTORC2 – mTOR Complex 2; NF-κB – nuclear factor kappa-light-chain-enhancer of activated B cells; NFAT – nuclear factor of activated T cells; p38MAP – p38 mitogen-activated protein kinase; PRC2 – polycomb repressive complex 2; Rictor – Rapamycin-insensitive companion of mammalian target of rapamycin; RelB – NF-κB subunit proto-oncogene RelB; RORγt – Retinoic acid-related orphan receptor gamma t; Satb1 – Special AT-rich sequence-binding protein 1; STAT – signal transducer and activator of transcription; TCR – T-cell receptor; TGFβR1 – transforming growth factor beta-receptor 1; Th17 – T helper 17 cells.

The above-mentioned mechanisms are summarized in **Figure 3**.

### 2.2 Epigenetic reprogramming of T cells in autoimmune diseases

To sum up as simply as possible, the thymus period of T-cell development and maturation includes both positive and negative selection, in which TCR signaling is the major checkpoint (46, 121). T cells expressing TCRs with a low affinity for self-peptide MHC complexes are subjected to differentiation into mature immunocompetent T cells (positive selection), whereas T cells expressing TCRs with a high affinity for self-antigens (self-reactive T cells) undergo negative selection *via* apoptosis (42, 45). As a result of such selection, only T-cells demonstrating autotolerance survive. Next, these naïve mature T cells, between maturity and activation, move to secondary lymphoid organs, such as the spleen and lymph nodes, including the tonsils and gut-associated lymphoid tissue. This is where they become activated after interaction with foreign peptides presented by the MHC molecules of antigen-presenting cells (APCs), such as macrophages, dendritic cells, and B cells. Thus, to participate in an adaptive immune response, a naïve T-cell must first encounter an antigen and then be induced to proliferate and differentiate into cells capable of contributing to the removal of the antigen (45, 47). Understandably, TCR signaling dysregulation can lead both to a state of near total/total immunologic unresponsiveness named anergy and autoimmunity in the case of impaired negative selection and intolerance of autoantigens. The tissue specificity and progression of T-cell-mediated autoimmunity are dictated in part by the repertoire of TCRs (46).

Although aberrant TCR signaling underlies autoimmunity, it is worth looking at epigenetic cell reprogramming, not only in terms of TCR function but also in a broader sense, considering other properties of T cells.

Histone modification and DNA methylation play important roles in the activation of naïve CD4+ and CD8+ T cells in the presence of specific cytokines with subsequent differentiation into effector or memory cells, and in the case of CD4+ T cells, adoption of distinct T helper fates. Activated naïve CD4+ T cells are highly plastic cells that can differentiate into various Th-cell fates characterized by the expression of effector cytokines such as IFN-γ (Th1), IL-4 (Th2) or IL-17A (Th17). Thus, epigenetic modifications greatly influence the functional differentiation of T-cell subsets, including linage commitment to short-lived effectors, long-term memory T cells, T regulatory cells, and other specific T-cell populations (21). The pattern of DNA methylation at key cytokine (IL-2, IL-4) loci influences the control of CD4+ T-cell differentiation and postthymic T-cell maturation (122). It was demonstrated that CD4+ Th-cell differentiation is modulated by lysine methyltransferase (KMT) Dot1 l-dependent dimethylation of lysine 79 of histone H3 (H3K79me2), which assures lineage-specific gene expression (123). Loss of Dot1 l (mediated by transgene Cd4-Cre, which becomes active in thymocytes at the DP stage) produces augmented expression of Th1-specific genes and excessive production of IFNγ at the expense of Th2 cell development. These events may confirm a central role of Dot1 l in Th1-cell lineage engagement and stability (123). Th1 and Th17 cells are involved in the pathogenesis of organ-specific autoimmune disorders, Crohn’s disease, Helicobacter pylori-induced peptic ulcer, acute kidney allograft rejection, and unexplained recurrent abortions (124, 125).. Moreover, numerous studies have found that the single type II IFN, IFN-γ, plays an essential role in the development and severity of systemic autoimmunity, particularly SLE (126). Dysregulation of KMT Dotl1 (KMT4), resulting in the shift of the Th1/Th2 balance paradigm toward Th1 and IFNγ overproduction, may promote autoimmune responses (127, 128). The role of DNA methylation is also significant in the plasticity of the Th17 subset, and under normal conditions, Th17 and naïve CD4 T cells had a similar methylation profile (129, 130). In addition, multiple studies have confirmed the ability of Th17 cells to convert into other CD4+ T cells in the presence of IL-12, both *in vitro* and *in vivo*, including conversion into a functional Th1-cell-like phenotype producing IFN-γ and lacking IL-17A secretion (131, 132).

Maintaining homeostasis and self-tolerance is inherently related to the function of Tregs (formerly known as suppressor T cells) because this specialized subpopulation of T cells can inhibit T-cell proliferation and cytokine production, playing a critical role in preventing autoimmunity (133). The transcription factor forkhead box protein 3 (Foxp3) is an essential molecular marker of Treg development in different microenvironments, and Foxp3 upregulation takes place either in the thymus (tTregs) or periphery (iTregs). Sustained expression of Foxp3 that assures balanced phenotypic plasticity and stability in Tregs requires both fine-tuned transcriptional and epigenetic events (133, 134). Recent reports have suggested that specific modifications of DNA and histones in the regulatory regions of the Foxp3 locus are key determinants for the establishment of the chromatin structure in conventional CD4+ T (Tconv) cells for their future differentiation into the Treg cell lineage (135, 136). In addition to the Foxp3 promoter, the three conserved noncoding DNA sequence (CNS) elements within the Foxp3 locus, i.e., CNS1, CNS2, and CNS3, are also targets of several modifying enzymes and are epigenetically regulated at different stages of Treg development (137). Defective Foxp3 expression involving abnormal Treg development and function may predispose patients to several autoimmune diseases (138). Decreased Foxp3 expression counteracts the suppressive effects, which are tightly regulated by Foxp3 itself and by its cooperation with several cofactors (139, 140). Foxp3 interaction with other transcription factors (e.g., GATA3 – member of the GATA family of conserved zinc-finger transcription factors, NFAT – nuclear factor of activated T cells, Runx – Runt-domain transcription factors, and STAT-3 – signal transducer and activator of transcription 3) may trigger either silenced or augmented gene expression (74, 139, 141). Therefore, epigenetic events that impair Foxp3 expression lead to disinhibition of the immune system with subsequent T-cell mediated autoimmunity (142). In other words, depending on the environment, Tregs gain effector functions upon loss of Foxp3 expression (143).

Posttranscriptional modulation of Foxp3 may be mediated by microRNAs (miRNAs), small single-stranded noncoding RNA molecules (containing approximately 22 nucleotides), which thus take part in epigenetically establishing Treg biological properties in health and disease (144, 145). After their posttranscriptional maturation, miRNAs are loaded into the ribonucleoprotein complex, i.e., RNA-induced silencing complex (RISC) modulates gene expression by binding to the 3’ untranslated region (UTR) of their target mRNAs through base-pairing, which in turn triggers mRNA degradation or translational inhibition (146). Computational estimates suggest that each human miRNA targets between 100 and 200 messages, usually in the 3′- UTR of the mRNA. Over 700 miRNAs are encoded in the human genome, and approximately one-third of all human genes are believed to be under the regulatory control of a miRNA (147). In relation to Tregs, there is mounting evidence that miRNAs regulate the proliferation, differentiation, and apoptosis of these T-cell subpopulations. Blockade of miRNA maturation in mice caused a lymphoproliferative phenotype similar to that observed in the absence of Foxp3 (148). Similarly, selective miRNA disruption in Tregs leads to uncontrolled autoimmunity (145, 149). For example, deletion of miR-146a-5p results in a breakdown of immune tolerance and the development of a fatal spontaneous autoimmune disorder due to inadequate inhibitory function of Tregs (150, 151). Suppression of the specific genes by Foxp3 may involve both direct binding to genetic regulatory elements and induction of miRNAs that specifically target the 3′-UTR of the same genes. Such coordinated action was demonstrated between Foxp3 and miR-155-5p in blocking the special AT-rich sequence binding protein 1 (Satb1) gene and zinc finger E-box-binding homeobox 2 (Zeb2) gene (152–154). With reference to miR-31, miR-24, and miR-210, there are grounds for assuming the possibility of direct action on the Foxp3 3′- UTR with subsequent reduction of Foxp3 expression levels and Treg phenotypic adjustment (155, 156). Another possibility of miRNA action on Tregs that leads to indirect reduction of its transcriptional activity includes interfering with the expression of proteins that cooperate with Foxp3, e.g., Eos (lkzf4), a member of the Ikaros family of transcription factors, interferon regulatory factor (Irf), or Satb1. This type of action is shown by miR-17, an individual mature miRNA of the miR-17-92 cluster (157). In turn, overexpression of miR-15a/16 contributes to the modulation of methylation/demethylation dynamics within the Foxp3 locus that influence Foxp3 expression (158).

Epigenetic modulation of Foxp3 expression also occurs at the protein level *via* covalent posttranslational modifications, including ubiquitination, acetylation, and phosphorylation of different amino acids (159–161). These processes influence Foxp3 subcellular localization, functional activity, and interaction with other proteins, mainly transcriptional activators or repressors. Thus, the resulting reduction in Foxp3 expression and corresponding reduction in suppressive Treg activity may promote autoimmune disorders (134, 135, 162).

Epigenetic alterations such as DNA methylation and histone modifications can regulate gene expression in mature T cells, with the possibility of dysregulation in autoimmune diseases. For example, in patients with SLE, numerous genes, such as CD11a (ITGAL), perforin (PRF1), CD70 (TNFSF7), and CD40LG (TNFSF5), in T lymphocytes were hypomethylated (163, 164). Several studies on the role of miRNAs in SLE revealed that decreased expression of DNMTs in CD4+ T cells of SLE shows correlation with three microRNAs (miR-21, miR-148a, and miR126) regulated by methylation (165). In addition to miRNAs, long noncoding RNAs (lncRNAs), defined as ≥200 base pairs in length with little or no translation potential, play a key role in imprinting control, immune cell differentiation, apoptosis, and immune responses. Many potential lncRNAs have been revealed to contribute to a new layer of molecular regulation of systemic lupus erythematosus (SLE) (166). LncRNAs play an indispensable role in SLE by interacting with proteins, DNA, and even RNA. Aberrant levels of NEAT1, Gas5, Lnc-DC, Linc0949, linc0597, MALAT1, and TUG1 are involved in the pathogenesis of SLE (167, 168). It was recently demonstrated that the novel lncRNA lincRNA00892 activates CD4+ T cells in SLE by regulating CD40 L, a 261-amino-acid membrane glycoprotein expressed on activated CD4 lymphocytes (169). Variation in the expression of noncoding RNAs (ncRNAs), both miRNAs and lncRNAs, interacting with the immune function of T cells influences susceptibility to SLE and the clinical course of this disorder (170).

Epigenetic reprogramming of T cells caused by ncRNAs is under intense scrutiny in relation to other autoimmune diseases, such as rheumatoid arthritis, systemic sclerosis, Sjogren’s syndrome, and organ-specific autoimmune diseases (e.g., autoimmune thyroid diseases and type 1 diabetes). The results of such investigation resemble those for SLE, however, with other ncRNAs (171–174).

## 3 The association between endometriosis and autoimmune diseases

The definition and basic characteristics of endometriosis with reference to its autoimmune linkages are briefly presented in the introduction (Chapter 1.). The reason for these links is unclear, but it might exist because mature endometriotic foci initiate inflammation, which may contribute to an imbalanced immune response inducing autoimmunity (175, 176). On the other hand, the abnormal immune response that occurs in endometriosis may be due to an already existing autoimmune disorder with a disturbed cytokine profile, altered cell apoptosis, and imbalances in immune cell function (177). The evidence is not clear as to which condition causes the other. Thus, there is still no conclusive cause of endometriosis, and researchers do not yet know what triggers the condition (178, 179). As already mentioned in the introduction, this chronic, progressive condition is not currently classified as an autoimmune disease (180). This may be because - at least initially - the immune system fails to recognize and target endometrial tissue growing elsewhere (ectopically) in the body. Endometrial foci themselves may have some ability to evade the immune response – similar to some cancers – by tricking or confusing immune cells that would otherwise attack those cells that form the lesions. Such a failure to recognize and target endometriotic foci may indicate that endometriosis is an immune disease with a deficit of immune recognition but not necessarily an autoimmune condition (181–183). Possible autoimmune pathogenesis of this proliferative disease may be supported by the fact that women with endometriosis may also have an increased risk of autoimmune comorbidities (e.g., SLE, RA, SS, MS, IBD) compared to healthy controls (36). In addition, endometriosis is more severe in patients who are also affected by autoimmune disease (184). As the pathogenesis of endometriosis continues to reveal itself, more autoantibodies are being discovered, and they may offer useful noninvasive tools for early diagnosis of endometriosis. This is important because diagnosis of ectopic dissemination of endometrial cells is usually delayed by an average of 8 to 11 years, leading to significant consequences in terms of disease progression (185). Various anti-endometrial antibodies may be used for early diagnosis in minimal to mild endometriosis, especially anti-SLP2, anti-TMOD3, anti-TPM3, and anti-PDIK1 L. Other nonanti-endometrial antibodies, such as anti-IMP1, anti-CA, aCL, and anti-STX5, may be used as additional noninvasive diagnostic tools (180, 185).

However, one should not forget that in the etiopathogenesis of endometriosis, hormonal disorders play an important, if not the most important, role. The disease is known as an estrogen-dependent and progesterone (P4)-resistant process (186, 187). In contrast to endometriosis tissue, estrogens are not locally produced in the endometrium. Several causes of P4 resistance in the endometrium have been postulated, including congenital “preconditioning”, whereby the *in-utero* environment renders infants susceptible to neonatal uterine bleeding and endometriosis (188). P4 action is crucial to decreasing inflammation in the endometrium, and deviant progesterone signaling results in a proinflammatory phenotype. Interestingly, chronic inflammation itself can induce a P4-resistant state (32, 189). The importance of excess estrogen exposure and P4 resistance in epigenetic homeostasis failure in endometrial/endometriotic tissue is crucial. Epigenetic alterations regarding transcription factors of estrogen and P4 signaling pathways in mesenchymal stromal cells (MSCs) are robust in endometriotic tissue (38). It is therefore logical that, unlike in autoimmune disorders where targeted immunosuppression is a priority, the treatment in endometriosis concerns hormonal imbalances and is primarily aimed at decreasing the endogenous ovarian production of estrogens (190, 191). In general, it is now appreciated that estrogens, and in particular E2, can control proinflammatory signals/pathways (192). The anti-inflammatory effects of estrogens are associated mostly with signaling *via* ERα and GPER, whereas even if not without controversy, an increased ratio of ERβ is associated with proinflammatory signatures (193–195). Variations in the expression of different estrogen receptor types may lead to some discrepancies in understanding the effects of estrogen on the immune system in health and endometriosis (192, 196). Markedly higher levels of ERβ and lower levels of ERα in human endometriotic stromal cells corresponds to EnSCs compared with EnSCs within eutopic endometrial tissues were reported (197, 198). Such overexpression of ERβ in endometriosis was associated with abnormally lowered methylation of a CpG island in the promoter region of the ERβ gene (ESR2) (199). High levels of ERβ, in turn, suppress ERα expression and the response to E2 in endometriotic stromal cells by binding to nonclassical DNA motifs in alternatively used ERα promoters (28). Lowered expression of ERα demonstrated in endometriosis may cause insufficient responsiveness to E2 with respect to progesterone receptor (PR) expression, leading to secondary P4 receptor deficiency and P4 resistance, which is commonly observed in women with this disorder (28, 188). In addition to DNA methylation, the epigenetic nature of the interaction between immune and hormonal systems that significantly impact endometriosis pathogenesis and development by modulating the immune response *via* estrogen and P4 receptors also encompasses noncoding RNAs: miRNAs (e.g., miR-148a, miR-30b-5p, miR-487a-5p, miR-4710, miR-501-3p, miR-378 h, and miR-1244) and lncRNAs (e.g., HOX antisense intergenic RNA - HOTAIR) (200–206). In the spectrum of consequences of hormonal profile modulation by epigenetic factors in endometriosis, the altered function of immune cells, including T cells, deserves attention (177, 207).

### 3.1 The immune landscape in endometriosis

Naturally, T cells do not function in isolation from the environment and changes in their environment may play a key role in the etiopathogenesis and course of endometriosis. This applies primarily to the endometriotic lesions and eutopic endometrium but also – although to a different extent – may be the result of an interaction with all of the cells in the body within reach of T cells. Moreover, the immune landscape in endometriosis is largely modulated by epigenetic factors (208). For example, aberrant DNA methylation patterns were demonstrated in the eutopic endometrium in endometriosis patients compared to the eutopic endometrium of endometriosis-free women (209). The level of DNA methylation in the whole genome was also different when comparing endometriotic stromal cells with the endometrium of healthy women. The observed differences in the methylation of the 403 genes examined pertained mainly to those encoding transcription factors, HOXA genes, and genes of nuclear receptors (210). The altered pattern of DNA methylation/demethylation within endometrial stromal cells translates to the upregulation or downregulation of specific proteins. Thus, endometriosis phenotypes are associated with specific proinflammatory and tissue remodeling cytokine profiles influencing the T-cell immune response. In addition, the DNA methylome is responsible for the overexpression of the genes encoding deoxyribonucleic acid methyltransferases DNMT1, DNMT3A, and DNMT3B in ectopic endometrium (209, 211, 212). Their expression levels were positively correlated with each other. Moreover, it was demonstrated that hypermethylation was confirmed only for the DNMT3A transcript but not for DNMT1 and DNMT3B transcripts in endometriotic stromal cells (213). Conversely, a significant reduction in the expression of DNMTs was found in other studies comparing the tissue obtained from endometriotic foci with endometriosis-free control specimens (213, 214). An estrogen-dependent and progesterone (P4)-resistant environment was created at the level of endometriotic stromal cells by the altered expression of estrogen receptor-ß (ERß) and P4 receptors (PRs) concomitantly with an epigenetic switch in GATA transcription factor isoform expression. This switch replaces GATA2, which is expressed in normal healthy endometrium, with GATA6 in endometriotic cells and appears to strongly contribute to the endometriotic phenotype (210, 215). Altered endometrial DNA methylation in endometriosis was most evident in the mid-secretory phase (P4 peak), where a bias toward methylation of CpG islands may lead to the disruption of the normal pattern of cycle-dependent DNA methylation modifications. Considering this, wide-range abnormalities of the chromatin remodeling machinery in endometriosis should become a logical consequence (38, 216).

Modulation of gene expression through histone modifications has been confirmed recently in endometriosis in relation to both eutopic and ectopic endometrial/endometriotic tissues. Profiles of normal and aberrant histone lysine methylation or acetylation patterns were analyzed intensively in animal and human endometrial tissue models (217, 218). In human samples, endometriotic foci are more hypoacetylated at H3 (but not at H4) compared to eutopic endometrium from healthy women. The endometriotic tissue was characterized by significantly lower levels of H3K9ac and H4K16ac compared to eutopic endometrium from patients and controls (219). The use of chromatin immunoprecipitation (ChIP)-polymerase chain reaction made it possible to demonstrate hypoacetylation of H3/H4 within the promoter regions of candidate genes that are recognized as downregulated in endometriosis (e.g., HOXA10, ESR1, CDH1, and p21WAF1/Cip1) when comparing endometriotic lesions and control endometrium (219, 220). The stereoidogenic factor 1 (SF1) promoter region was enriched for acetylated H3 and H4 in endometriotic vs. control endometrial tissues. This acetylation was correlated with the increased expression of SF1 in the lesions. In addition to altered activities of HDACs, hypermethylation at H3K4, H3K9, and H3K27 was demonstrated in endometriosis (218, 219).

Differences in the expression of over 100 miRNAs have been reported in endometriotic cells. Such miRNA profiling may play a pivotal role in the development of miRNA signatures for endometriosis and expand our knowledge on the roles of individual miRNAs in the pathomechanism of this disease (220).

Thus, when looking at a slightly more general perspective of the role of T cells in endometriosis, local endometrial function/dysfunction, including cell proliferation, inflammation, immunogenicity of endometriotic stromal cells, angiogenesis, and sex steroid hormone responsiveness, may be relevant. Regardless of whether the observed disorders are caused by epigenetic factors or DNA sequence changes, the immune landscape in endometriosis interacts with T cells through overproduction of prostaglandins (PGE2), metalloproteinases (MMP-2, -3, -9), cytokines (TNFα, IL-1β, IL-8, IFNγ, MCP-1, and MIF) and adhesive molecules (VCAM-1, ICAM-1) (221). Furthermore, reactive oxygen and nitrogen species (ROS/RNS) production induced by pathological conditions influences aerobic glycolysis in activated CD4+ T cells and has an immunomodulatory effect on the mechanisms of antigen presentation and T-cell receptor signaling (222, 223).

The use of whole-tissue deconvolution with single-cell transcriptomic (scRNAseq) analysis made it possible to create an atlas of the human endometrium during the menstrual cycle (224, 225). Such a high-resolution molecular and cellular characterization of the human endometrium as a dynamic tissue that undergoes cyclic changes provides new opportunities to study the pathophysiology of endometriosis, including the cellular complexity of disease development. scRNAseq analysis can provide insights into the phenotypes of endometrial/endometriotic cell populations (226). Moreover, the full complement of immune and nonimmune cell types contributing to a proinflammatory background can be precisely identified across the menstrual cycle (227, 228). For example, subpopulations of fibroblasts related to endometriosis development were identified (228).

The coexistence of some diseases, including autoi-mmunological ones, may significantly change the influence of environmental factors on T cells. It was recently established that the presence of concomitant autoimmunity is associated with an advanced stage of disease in women with endometriosis. Although without a known explanation, this does indicate the possibility of a more aggressive course of the disease in patients who are also affected by autoimmune disturbances (184).

### 3.2 Epigenetic Reprogramming of T Cells in Endometriosis

There is substantial evidence of aberrant function of almost all types of immune cells in women with endometriosis, including altered T-cell reactivity and NK cytotoxicity, polyclonal activation of B cells and increased antibody production, increased number and activation of peritoneal macrophages, and changes in inflammatory mediators (175, 229–232). As in the title of this chapter, T-cell disorders in endometriosis are discussed. In contrast to macrophages, dendritic cells, and toll-like receptors (TLRs), which are integral components of the innate immune system, Th (Th1/Th2/Th17) cells and Tregs are the main components of the adaptive immune system. The rationale for a potential role of T cells, especially Tregs, acting either alone or in combination in the initiation, maintenance, and progression of endometriosis is because the growth and progression of endometriosis continues even in ovariectomized animals. Thus, in addition to ovarian steroid hormones, the establishment and growth of endometriotic foci outside the uterine cavity can be regulated by the innate/adaptive immune system (175, 233, 234). Interpretation of the results of many comparative T-cell studies in women with endometriosis vs. normal (control) women is difficult because most of the research was carried out under different (incompatible) conditions, using small and not precisely defined groups/subgroups of patients. Identification of T-cell subtypes typically took place in the blood, peritoneal fluid, or endometrial/endometriotic foci (235). In connection with the pursuit of greater selectivity T-cell markers are also changing. For example, currently, in both mice and humans, the markers of Tregs are often presented as CD4^+^CD25 ^high^ CD127 ^–^ Foxp3^+^, where CD25 and Foxp3 are constitutive markers appropriate for isolation of Tregs, and CD127 expression is inversely correlated with both Foxp3 expression and related CD4^+^Tregs immunosuppressive function (236, 237). Previously, such a set of markers was not obvious, which makes the interpretation of the results over many years difficult. After considering the above reservations, which may explain some discrepancies and controversies, it is assumed that there are quantitative and qualitative changes in the T-cell population in endometriosis. Loss of balance between Th1/Th2/Th17 and Tregs leads to inappropriate secretion of T-cell-related cytokines (abnormal cytokine profile) and inflammation that induces progression of endometriotic lesions (238). Regarding T-cell subpopulations, it was demonstrated that the CD4+ T-cell profile in lesions and peripheral blood is altered in women with endometriosis. The proportion of Th1 lymphocytes was significantly lower in endometriotic lesions than in eutopic endometrium, and the Th17 lymphocyte fraction was significantly higher in the lesions than in eutopic endometrium. In addition, in peripheral blood, the Th1-cell fraction was significantly higher in patients with endometriosis than in women without the disease (231, 239). Posttranslational modifications (PTMs) are key molecules involved in Th17/Treg differentiation and function (Foxp3, RORγt, and STATs), regulate the Th17/Treg balance, and initiate autoimmune diseases caused by dysregulation of the Th17/Treg balance. An epigenetic toolkit contains modulators of genome architecture such as phosphorylation, methylation, nitrosylation, acetylation, glycosylation, lipidation, ubiquitination, and SUMOylation. Phosphorylation is the most common PTM contributing to Th17/Treg cell functions, whereas interactions between multiple PTMs influence Th17/Treg differentiation (97, 240, 241).. An increased number of Tregs has been reported in specimens (e.g., eutopic endometrium, peripheral blood, and peritoneal fluid) obtained from women with endometriosis compared to endometriosis-free control women (242–244). It was postulated that such an increased amount of Tregs may modulate the inflammatory response toward the establishment of an anti-inflammatory environment by suppressing activation of the immune system evoked by the endometriotic foci. Consequently, a reduced immune response enables ectopic endometrial implantation and propagation, resembling immune tolerance in allogeneic grafts and pregnancy (245). It can also be the opposite: Tregs could be moving toward the ectopic endometrial focus to reduce the severe inflammatory reaction (246). Thus, a higher frequency of circulating Tregs in patients with endometriosis compared with controls may be considered a compensatory mechanism to regulate the inflammatory condition in this disease (247).

There is also no doubt that, in addition to their immunoregulatory role, Tregs are involved in both normal and pathological angiogenesis. The association between angiogenesis and Tregs can be viewed in terms of either relation to the vascular endothelial growth factor (VEGF) signaling pathway or mediation *via* modulation of other immune cells and their release of cytokines and growth factors that influence angiogenesis (248). Interestingly, the role of Tregs in angiogenesis has been shown to be highly tissue- and context-specific and, as a result, can yield either pro- or antiangiogenic effects. This also pertains to different stages of endometriosis; however, it makes prediction unreliable (249–251).

CD4+ CD25+ Tregs (or even more precisely characterized in the current papers as CD4+CD25 ^high^ CD127 ^–^ Foxp3^+^ cells) are mainly produced in the thymus from where they migrate to the circulation as natural Tregs (nTregs), and a much smaller subpopulation differentiates in the periphery from naïve T cells into induced Tregs (iTregs) (252, 253). It has been shown that concurrent induction of Treg-specific epigenetic changes and the expression of transcription factor Foxp3 controlling a substantial part of Treg development and function is crucial for lineage specification and functional stability of Treg cells (254). Treg deficiency or dysfunction exaggerates local inflammation and angiogenesis and simultaneously facilitates the attachment and growth of endometrial implants (250).

The altered immune response in endometriosis may be attributed to defective apoptotic processes. Increased concentrations of cytotoxic (CD8+) T lymphocytes (CTLs) and HLA-DR- activated T cells were observed in peripheral blood during the luteal phase compared to the follicular phase of the menstrual cycle in healthy women, whereas women with endometriosis did not exhibit fluctuations in the concentrations of cytotoxic and activated peripheral blood lymphocytes during the menstrual cycle. In addition, a marked increase in Treg concentration, which was positively correlated with the serum levels of cortisol, was detected in the peripheral blood of women with endometriosis only (255). The cytoplasmic granules of CTLs contain perforin, a cytolytic mediator that may induce apoptosis, because they form pores when inserted into the target cell membrane (256). A significant reduction in the cytotoxic potential of CTLs was demonstrated in endometriosis, where the number of perforin^+^ CTLs among CD8^+^ T cells in the menstrual effluent was decreased compared to healthy controls. Perforin mRNA levels correlate with the methylation status and accessibility of the promoter at the 5′ flanking region of its gene. Thus, the defective apoptotic process may be caused by DNA hypermethylation and changed chromatin structure affecting negatively perforin gene expression in T cells (257).

At the same time, a decreased concentration of intercellular adhesion molecule-1 (ICAM-1) was observed in the serum of endometriosis patients. The transmembrane glycoprotein ICAM-1 plays a role in inflammatory processes and in the T-cell-mediated host defense system. ICAM-1 is constitutively expressed over the cell surface and its expression can be modulated by transcription and epigenetic factors related to cellular stress, proinflammatory cytokines, and viral infection (258). It functions as a costimulatory molecule on antigen-presenting cells to activate MHC class II restricted T cells and on other cell types in association with MHC class I to activate cytotoxic T cells. Deletion of the 5′ flanking region of ICAM-1 gene at positions -329 and -485 upregulates the basal level expression of ICAM-1, suggesting the presence of a regulatory silencer within this region (259).

Both fewer perforin+ CTLs and a reduced concentration of ICAM-1 may reflect a reduced capacity to remove endometrial cells from ectopic locations (260–262). ICAM-1 alone or together with soluble vascular cell adhesion molecule 1 (VCAM-1) may be a promising biomarker for diagnosing endometriosis. However, according to the results of a meta-analysis, ICAM-1 used alone has moderate diagnostic accuracy, while for unknown reasons, the diagnostic accuracy is higher in patients of Asian ethnicity than in those of Caucasian ethnicity (263, 264).

Because of the opposite effect on the immune response, proinflammatory Th1 and Th17 cells should be balanced by Treg subsets with anti-inflammatory capacity. An important element of such a balance is a specific cytokine profile with IFNγ and IL-2 produced by Th1, IL-17A synthetized by Th17 and IL-10 and transforming growth factor (TGF)-β secreted by Tregs (265–268). It was documented that increases in the level of IL-17A and the presence of Th17 in peritoneal fluid correlate positively with the severity of endometriosis and infertility associated with this disorder (269, 270). The number of Th17 cells in peritoneal fluid was higher in severe endometriosis (stages III and IV) than in early/not advanced (stages I and II) endometriosis (270, 271). IL-17A may play a role in the development of endometriosis by stimulating inflammatory responses, angiogenesis, and proliferation of endometriotic stromal cells (272, 273).

An imbalance between the cytokine profile related to Th1 and Th2 responses was suggested in the etiopathology of endometriosis. The shift toward the Th2 immune response component (a reduced Th1/Th2 ratio among T cells in the peritoneal fluid) with the relative increase in cytokines, characteristic of this pattern of immune response (IL-4, IL-5, IL-10, and IL-13) should be considered. These cytokines are associated with the promotion of IgE and eosinophilic responses in atopy and interleukin-10, which has more of an anti-inflammatory response. In excess, Th2 responses counteract the Th1-mediated perpetuating autoimmune responses (274). Indeed, in endometriotic lesions, the levels of IFN-γ and IL10 and the ratios of IL4/IFN-γ, IL4/IL2 IL10/IFN-γ, and IL10/IL2 are significantly elevated in the peritoneal fluid of endometriosis patients compared to healthy controls (238, 275). For example, the release of IL-4 from Th2 cells may lead to a dose-dependent increase in the expression of 3β-hydroxysteroid dehydrogenase (HSD3B2) mRNA, a pivotal enzyme for estrogen production (276). Therefore, endometriosis progression may be stimulated by an IL-4 dependent, local increase in estrogen concentration. Moreover, IL-4 promotes the proliferation of endometriotic stromal cells (ESCs) and endometriosis progression by activating p38 mitogen-activated kinases (p38 MAPKs), stress-activated protein kinase/c-Jun kinase and p42/44 MAPK. All these enzymes are strongly induced *in vivo* by environmental stresses and inflammatory cytokines (277). Activity of the transcription factor GATA binding protein 3 (GATA3) is regulated by estrogen, and their synergistic action regulates Th2 cytokine (e.g., IL6, IL8, and IL10) expression in endometrial cells (both eutopic and ectopic). Therefore, GATA3 integrates estrogen signaling to induce Th2 cytokine expression in endometriotic lesions, thereby promoting endometriosis progression (278). Interestingly, eutopic endometrial tissues from patients with endometriosis have higher mRNA levels of GATA-binding protein 3 (GATA3) compared to normal endometrial tissue (279). Because the development and maintenance of endometriosis highly depends on the estrogen pathway, overexpression of the two proteins that control key steps of 17β‐estradiol biosynthesis, steroidogenic acute regulatory protein (StAR) and aromatase (CYP19), may contribute to formation of ectopic lesions (280). Hypomethylation of the promoter and/or intronic regions of StAR and CYP19 was shown to cause their incorrect expression in ectopic foci (281, 282). Hypomethylation was also detected within the promoter and/or intronic regions of several aberrantly expressed nuclear receptors that mediate the effect of steroid hormones or modulate steroidogenic activity (e.g., estrogen receptor β, steroidogenic Factor 1 (SF-1)) (283, 284). These data indicate that DNA methylation is coordinately regulated to facilitate production or to enhance activity of 17β‐estradiol (E_2_) in endometriosis (285). The central role of epigenetic regulation on the steroid hormone pathway manifests itself in two directions. This means that inactivation of E2 is also regulated by DNA methylation. In addition, conversion of E_2_ to less potent estrone (E_1_) is suppressed in endometriosis because the converting enzyme 17β‐hydroxysteroid dehydrogenase type II is inactivated in ectopic stromal cells due to hypermethylation (286).

Interleukin 6 (IL-6) promotes CD4+ Th2 differentiation by activating transcription mediated by nuclear factor of activated T cells (NFAT) and – at the same time – inhibits Th1 differentiation by interfering with IFN-γ signaling and expression of suppressor of cytokine signaling 1 (SOCS1) (287, 288). Increased IL-6 levels were demonstrated in ESCs isolated from women with endometriosis compared to healthy controls (289). IL-6 expression in endometriotic cells may be induced by IL-1β and TNF-α (290). According to recent studies, IL-6 pathway gene expression can be affected by DNA methylation, microRNAs, and posttranslational modifications (291, 292).

Interleukin 23 (IL-23) is a proinflammatory cytokine composed of two subunits, IL-23A (p19) and IL-12/23B (p40), produced primarily by activated macrophages and dendritic cells. It drives the differentiation and activation of T helper 17 (Th17) cells and maintains their phenotype, such as their cytokine production, including IL-17A, which is their major effector molecule (293). Well-established experimental data support the concept that defective IL-23/IL-17 axis activation contributes to the development of several autoimmune (e.g., IBD, RA, SS, MS) and inflammatory diseases, including endometriosis (294). Levels of IL23 were significantly higher in the peritoneal fluid of women with endometriosis than in normal controls (295). Activated naïve T cells produce IL23 and consequently increase production of IL10 and IL17, both of which are factors promoting endometriosis progression (296). It was recently documented that environmental factors may significantly contribute to activation, modulation, or dysregulation of the IL-23/IL-17 axis (297).

### 3.3 Normalization of T-Cell function as a target for novel epigenetic-based therapies

Despite the fact that the etiology of endometriosis is complex and multifactorial, an abnormal immune response in this disease with evident changes in T-cell activities clearly indicates the possibility of treating these heterogeneous cells as therapeutic targets (32, 133, 175, 235). The suggestions for potential therapeutic measures presented below are limited to epigenetically modulated T-cell disorders.

Unlike autoimmune diseases, in endometriosis, we are dealing with a deficit of immune recognition by T cells (176). Therefore, an augmented T-cell-dependent immune response may improve the elimination of ectopic cells within endometriotic foci (177). The cells can be influenced directly or indirectly. In the latter situation, and generally in the absence of a selective effect, one should consider the effects of epigenetic modulation with respect to other cells and organs, that is, systemic action. It is therefore important to be aware of the possibility of both the synergistic effects and the side effects when acting on other cells (e.g., ectopic and eutopic endometrial tissue). Increased activity of T cells may also adversely affect the course of autoimmune diseases frequently cooccurring with endometriosis (176).

There are three families of epigenetic proteins considered as susceptible to disease modifying drugs: readers, writers, and erasers (298). Initially, the readers recognize and bind to specific covalent DNA modifications, as well as histones, and non-histone proteins. Next, the writers introduce various chemical modifications on DNA and histones. Finally, the erasers are responsible for enzymatic removal of these biochemical tags. Therefore, all these stages of epigenetic modulation are druggable targets using small molecular-inhibitors (SMIs) including approved by US Food and Drug Administration (FDA) azanucleosides, vorinistat, fedratinib. These drugs targeting DNMTs, HDACs, and JAK2, respectively (299, 300). The significant advancement of work on other SMIs, including on-going clinical trials, causes the implementation of new drugs is a matter of time. However, at present the use of SMIs in clinical settings is mostly limited to hematological malignancies (300–302). Safety issues related mostly to the lack of selectivity produce significant limitations with implementation of SMIs. Cardiovascular, CNS, stem cell homeostatic, developmental and reproductive, transgenerational, and carcinogenic effects are among the potential consequences of targeting epigenetic mechanisms (303).

Naturally, at this stage of advancement of research on epigenetically targeting T cells in endometriosis, the rationale and their main directions are signaled. For example, to identify the epigenetic changes involved in endometriosis, a genome-wide analysis of DNA methylation and enrichment of H3K4me3 and H3K27ac histone marks in sorted CD4+ and CD8+ T cells may be performed (304). However, it is important to realize that only full knowledge about the etiopathology of endometriosis, and in this case, the importance of epigenetic interactions, will make it possible to increase the effectiveness of these activities oriented on therapy.

The drugs (SMIs) available on the market today carry too great a risk to women of childbearing age in regards to fertility and embryo-fetal development, leading to a pregnancy category D warning on the label (303). As it was mentioned elsewhere, up to 50% of endometriosis-related treatments have been performed in order to restore the ability to become pregnant (23, 24).

#### 3.3.1 Adjustment of hormonal imbalances

Considering that endometriosis is an estrogen-dependent and P4-resistant disorder, alignment of hormonal dysregulation is a widely used symptomatic treatment. Estrogen has been shown to modulate all subsets of T cells, including CD4+ (Th1, Th2, Th17, and Tregs) and CD8+ cells (305, 306). Human CD4+ T cells and CD8+ T cells express both estrogen receptors, ERα and Erβ, and are involved in the nongenomic G protein-coupled estrogen receptor (GPER) pathway (307–309). It was suggested that targeting abnormally lowered methylation of a CpG island in the promoter region of the ERβ gene (Esr2) may have promising therapeutic effects, reducing proinflammatory signatures of T cells. Moreover, the resulting decrease/normalization in ERβ expression may restore the normal ERα expression required for sufficient responsiveness to E2 with respect to progesterone receptor (PR) expression, which counteracts P4 receptor deficiency and P4 resistance. Alternatively, induction of hypomethylation in the respective CpG islands of the promoter regions of ERα and GPER may be used, as the anti-inflammatory effects of estrogens are associated mostly with signaling *via* ERα and GPER (164, 310).

In contrast to the mouse, most studies did not confirm the classical P4 receptors (PR-A and PR-B) in human T cells. However, P4 effects may be mediated *via* the two families of membrane PRs belonging to the progestin and adipoQ receptor (PAQR) family (also known as membrane progestin receptors (mPRs)) and the progesterone receptor membrane component (PGRMC) receptors. In humans, three members of the PAQR family (PAQR5, PAQR7, and PAQR8) and two members of the PGRMC family (PGRMC1 and PGRMC2) were identified. Progesterone modulates the pattern of T-cell cytokine production in a dose-dependent manner (311, 312). Hence, reverting relative P4 resistance may augment the potentially beneficial influence of these cytokines. Downregulation of PR-B due to promoter hypermethylation of PR-B was reported in the endometrium of women with endometriosis (313). Accordingly, with regard to T cells, P4 increases the number of CD4+ CD25+ FoxP3+ Tregs in the maternal-fetal interface of pregnant mice (314). It was demonstrated that Treg deficiency intensifies the course of endometriosis primarily regarding the intensity of inflammation (198, 315). Nevertheless, the recommendation of a particular course of action (i.e., methylation, demethylation) in relation to estrogen and P4 receptors in T cells must be preceded by comparative analysis with endometrial/endometriotic cells because the effects of interactions with these receptors may differ significantly.

In addition to methylation, noncoding RNAs: miRNAs (e.g., miR-148a, miR-30b-5p, miR-487a-5p, miR-4710, miR-501-3p, miR-378 h, and miR-1244) and lncRNAs (e.g., HOX antisense intergenic RNA - HOTAIR) are involved in the epigenetic spectrum of interactions between immune and hormonal systems in endometriosis (316, 317). Future studies are needed to determine whether estrogen and P4 receptors in T cells may be modulated efficiently by noncoding RNAs to restore the environment with normal responsiveness to estrogen and P4.

Another promising treatment option is based on the demonstration that the estrogen – indoleamine 2,3-dioxygenase-1 (IDO1) – mannose receptor C, type 2 (MRC2) axis participates in the differentiation and function of Tregs and is involved in development of endometriosis (318). In cocultured naïve T-cell-macrophage-endometrial stromal cells (ESCs), a specific blocker of IDO1,1-methyl-tryptophan (1-MT) produced a significant decrease in Treg differentiation, particularly in the IL-10+ Treg subpopulation. Therefore, 1-MT-pretreated ESC-educated Tregs exhibited impaired suppressive function. Moreover, estrogen promoted the differentiation of Tregs by elevating IDO1 expression in ectopic lesions (319). At the same time, expression of MRC2, which is an upstream molecule of IL-10 required for Treg differentiation in ectopic lesions (especially CD4^high^ Tregs), was significantly lowered. Thus, blockage of IDO1 in ectopic lesions, which does not influence the physiological functions of estrogen, may be considered a potential therapy for endometriosis (318).

#### 3.3.2 Influencing T-cell development, differentiation, and activation

Increasing data show that epigenetic reprogramming of T-cell development and differentiation can contribute to the development of new, breakthrough treatments in endometriosis (320). Quantitative and qualitative disorders in the T-cell population in endometriosis (vs. endometriosis-free T cells) that can now be compensated for under laboratory conditions by using epigenetic mechanisms include the Th1:Th17:Treg lymphocyte ratio and number, influencing T-cell-dependent apoptosis and angiogenesis, modulating Th2 cytokine expression and influencing Th17 activation (156, 242, 321–323).

The discovery of Foxp3 was a turning point in understanding the molecular determinants leading to the generation and maintenance of Tregs (82). Maintenance of Foxp3 protein expression in regulatory Tregs is crucial for a balanced immune response. Recent reports suggest that specific modifications of DNA and histones are necessary for establishing the chromatin structure in conventional CD4^+^ T cells (T conv) as a prerequisite for their future differentiation into the Treg cell lineage. Thus, Tregs support a distinct DNA methylation pattern compared to Tconv, and specific epigenetic mechanisms critically influence Foxp3 stability (324). Moreover, several studies have demonstrated that during inflammation (e.g., endometriosis-related), Treg cells may lose their phenotypic properties and be converted into effector T cells secondary to the alteration of Foxp3 expression and stability (325–327).

The results of animal studies have shown that Foxp3 expression is regulated by miRNA (96, 97). For example, deletion of miR-146a-5p results in a breakdown of immune tolerance and development of a fatal spontaneous autoimmune disorder, whereas Foxp3 acting together with miR-155-5p blocks key inducers of effector lineage commitment, such as Satb1 and zinc finger E-box binding homeobox (98–100, 328). For future therapeutic purposes, the most important thing is therefore that Foxp3 imposes a multilayered suppression of specific genes in Treg cells by both direct binding to genetic regulatory elements and by induction of miRNAs that specifically target the 3′ UTR of the same genes. Epigenetic machinery adjusting the Treg phenotype for medicinal purposes could be based on the action of miR-31, miR-24, and miR-210, which directly target the Foxp3 3′ UTR (103, 104). Regulation of Foxp3 expression and function may also take place at the protein level in the form of covalent posttranslational modifications, such as ubiquitination, acetylation, and phosphorylation of different amino acids. Following these processes, changes in the subcellular localization and activity of Foxp3 should be expected. Such effects resulting from different interactions with other proteins, mainly transcriptional activators or repressors, deserve attention from a therapeutic point of view.

**Figure 4:**
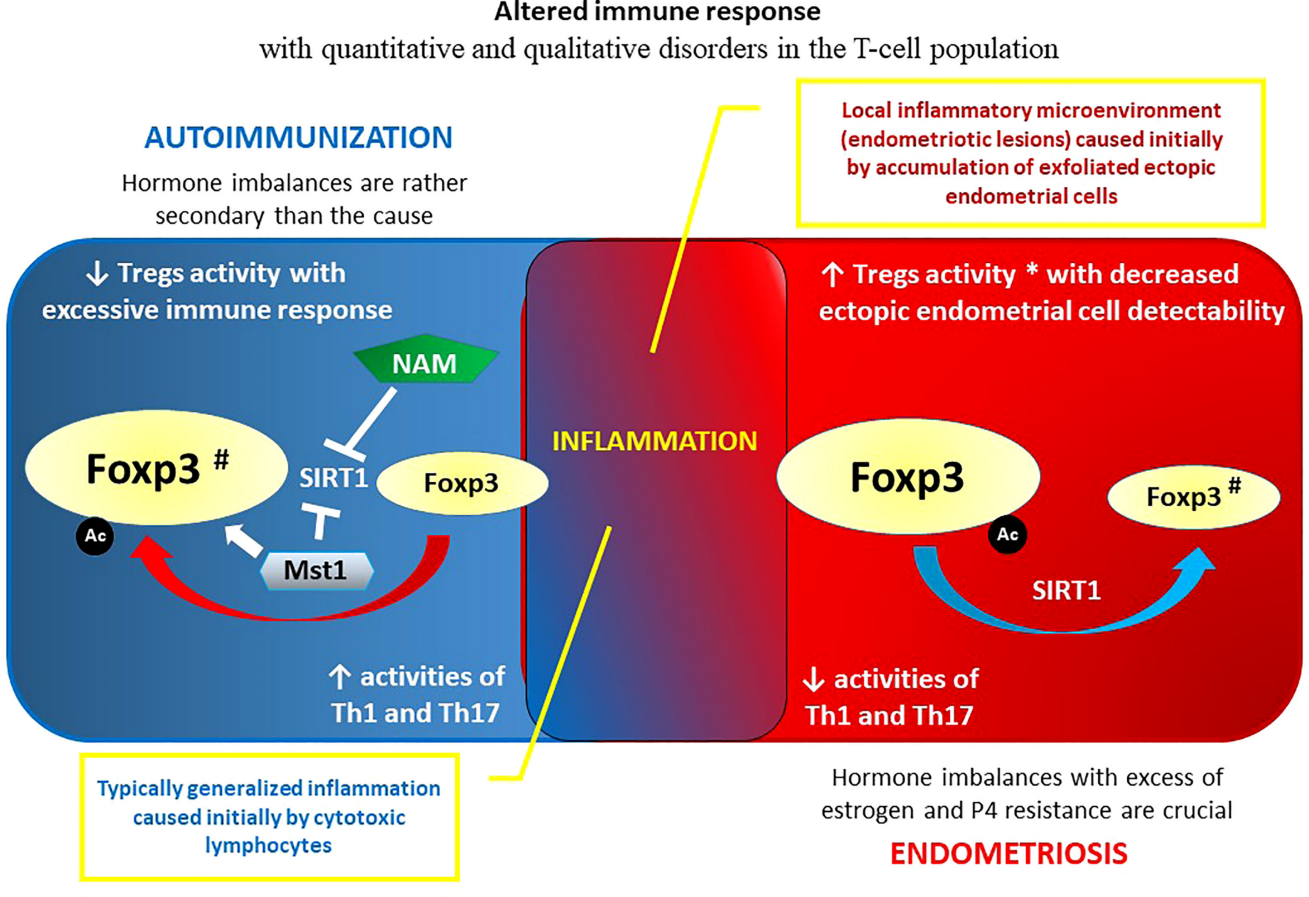
Regulatory T cells and altered immune response: autoimmune disease vs. endometriosis. Maintenance of Foxp3 protein expression in regulatory Tregs is crucial for a balanced immune response. The rationales for pharmacological modulation of SIRT1 activity in Tregs as an example of novel therapeutic approach for controlling immune responses in endometriosis and autoimmunity are also showed. **^#^** Restored to normal Foxp3 expression after the treatment; * It can also be opposite: increased number and activity of Tregs may be considered as an insufficient compensatory mechanism to overcome inflammation (194, 195). Epigenetics of Tregs appears to be a common denominator for autoimmunity and endometriosis. See the main text (Chapter 3. The association between endometriosis and autoimmune diseases) for details. Ac – acetylation; Foxp3 – forkhead box P3 protein; Mst1 – mammalian sterile 20-like kinase 1; NAM – nicotinamide; SIRT1 – sirtuin 1 (silent mating type information regulation 2 homolog 1); Th1 – T helper 1 cells; Th17 – T helper 17 cells; Tregs – regulatory T cells.

Sirtuin-1 (SIRT1)-mediated deacetylation of Foxp3 leads to its decreased expression as a result of increased ubiquitination and subsequent degradation in the proteasome (329). Conversely, application of nicotinamide, a SIRT1 inhibitor, reduces Foxp3 degradation together with increased Treg cell number and suppressive activity. These findings may indicate the central role for SIRT1 in the regulation of Foxp3 protein levels and thereby in the regulation of Treg suppressive capacity. Pharmacological modulation of SIRT1 activity in Tregs may therefore provide a novel therapeutic approach for controlling immune responses in endometriosis. This can be done by regulating mammalian sterile 20-like kinase 1 (Mst1), which increases Foxp3 acetylation and promotes its activity both indirectly, by inhibiting the activity of SIRT1, and directly, by interacting with Foxp3 and preventing its binding to SIRT1 (330). Interestingly, influencing the Foxp3 acetylation level deserves attention as a potential therapy aimed at restoring the normal balance between Treg and Th17-cell lineage differentiation (331). This is because the transcriptional coactivator with PDZ-binding motif (TAZ) that promotes differentiation toward Th17 and inhibits Treg development, regulates Foxp3 acetylation by competing with it for binding to TIP60, a nuclear histone acetyltransferase (HAT) that mediates Foxp3 acetylation and inhibits its proteasomal degradation (331). Thus, a decrease in Foxp3 acetylation constrains differentiation toward Tregs. Sirtuin-targeted treatment in altered immune response (autoimmune disease vs. endometriosis) aimed to restore optimal Foxp3 expression in Tregs is presented in **Figure 4**.

#### 3.3.3 Overview of T-cell reprogramming

T-cell reprogramming should be considered an effective measure to overcome the altered immune response in endometriosis, including that caused by epigenetic factors (332). Naturally, the crucial issue is comprehensive understanding of the etiopathogenesis of these disorders that present unclear association with autoimmunization. The question to be answered is as follows: should we potentiate or suppress T cells in endometriosis? More precisely: action of which T-cell subpopulations should be strengthened, and which should be inhibited? Only then can the course of action be correctly determined. Research on T-cell reprogramming in endometriosis is very intense and advanced; however, as opposed to T-cell-based immunotherapy in cancer, it has not yet been translated into clinical practice.

The TCR is sufficient to direct antigen-specific T-cell differentiation and redirect their cytotoxicity. Custom TCR reprogramming may revert the condition in which the immune system fails to recognize and target endometrial tissue growing elsewhere in the body. Now, TCRs can be engineered to possess more specificity, affinity, reactivity, and broad-spectrum binding ability. For instance, TCRs can be engineered to potentially recognize all peptides processed and presented in the context of MHC molecules, thus allowing TCRs to target both surface and intracellular antigens. Exodomains of the α and β subunits of the TCR can be modified by replacing their variable domains with antibody domains that can recognize endometriosis-associated antigens (333). T-cell receptor gene rearrangement may also provide a lower or higher sensitivity to gene silencing (e.g., in human CD8+ memory T cells).

Chimeric antigen receptors (CARs) are a class of synthetic TCR receptors that reprogram lymphocyte specificity and function. Designed to bind to certain proteins (e.g., expressed within endometriotic foci), CARs constitute an effective genetic optimization of T cells to redirect specificity. The use of CARs in the treatment of epigenetic modifications or environmental endometriosis-causing agents is only a matter of time.

Discovery of immune checkpoint inhibitors about ten years ago, and then the development of CARs, created new options for treating hematological malignancies (334, 335). Clinical trials employing CARs are also conducted in solid tumors, including gynecological cancers (336). There are undoubted parallels in the development between cancer and endometriosis. The way of mutagenesis, pelvic spreading, immunological adaptation, and difficulties in eradication justify to consider endometriosis as “a cancer of no kill” (337). Ultra-mutated phenotype of ectopic endometriotic cells requires an escape form the immune surveillance during the development of endometriosis. As in malignant tumors, the interaction TCR/antigen/MHC self-recognition complex and the PD-1/PD-L1 immune checkpoint must be abolished to avoid T cell-mediated cytotoxicity. This is achieved by upregulated expression of PD-1/PD-L1 in endometriosis (338). Accordingly, serum PD-1 level is positively correlated with endometriosis-related infertility (339). Such immune escape in endometriosis makes it possible to apply an immune checkpoint blockade therapy using CARs that has revolutionized cancer treatment. If in a wide range of cancers that are characterized by genomic hypermutation and a high replication error rate, targeting PD-1/PD-L1 was effective, endometriotic cells, with identical phenotype characteristics, may very likely respond similarly (340).

Both custom TCR reprogramming and CAR generation can be performed using *in vitro*-transcribed (IVT) mRNA. After selection of the target of the CAR T cells, IVT-mRNA is precisely positioned within the created CAR IVT-mRNA molecule. The transfection of the mRNA transcript into T cell must ensure optimal/efficient expression the CAR molecule on T cell surface (341). This method is characterized by rapid and facile production and an acceptable safety profile. Unlike DNA, IVT mRNA has no risk of causing insertional mutagenesis and no long-term concern for side effects because of its labile nature (342).

The inhibition of epigenetic regulators may also be skillfully used to obtain the desired functional profile of T cells suitable for treatment of endometriosis. Inhibitors of DNA methyltransferase (e.g., 5′-azacytidine), histone deacetylase (e.g., valproic acid), histone methyltransferase (e.g., BIX-01294), and histone demethylase (e.g., tranylcypromine) should be considered (343, 344). Remarkable developments in the basic understanding and tools for reprogramming have begun to show the clinical impact of cellular reprogramming. Thus, genetically, and epigenetically reprogrammed T cells hold great promise in the areas of immunotherapy, including endometriosis, offering great hope for curative responses in women with ectopic endometriotic lesions. Adoptive transfer of profiled Tregs is a promising new therapeutic option to treat detrimental inflammatory conditions after transplantation during autoimmune disease and endometriosis, including disorders caused by epigenetic factors (345).

## 4 Concluding remarks

Dysfunction of the immune system is the essence of both autoimmune diseases and endometriosis (346, 347). Therefore, the altered function of T cells in these disorders is the subject of intense research. Understanding the role of epigenetic factors and determining which T-cell functions are controlled epigenetically could lead to a breakthrough in the final elucidation of endometriosis etiopathogenesis (348). The prevailing view thus far is that, in contrast to autoimmune diseases, silencing the immune response prevents detection and destruction of the endometriotic foci (349). Changes in T-cell count and/or T-cell subtype proportion together with modulation of T-cell activity may be a therapeutic target in endometriosis that involves epigenetic mechanisms. However, except for observational, cross-sectional studies, it is difficult to perform reliable investigations on endometriosis patients. Most of the immunological endometriosis research is performed on animals. In nature, spontaneous endometriosis affects only those mammalian species that menstruate, including primates, some bat species, spiny mice, and elephant shrews (350, 351). Thus, many animal models are not fully reliable, since endometriosis is induced artificially and does not represent all the phenomena present in the disorder. Such artificial endometriosis behaves differently than spontaneous endometriosis, even in the same experimental animal model or in the same animal (352, 353). T-cell reprogramming creates a new therapeutic option that may be tested to a large extent without the exploitation of animal models. Since epigenetics appears to be a common denominator for hormonal and immunological aberrations in endometriosis, adjustment of hormonal imbalances and influencing T-cell development, differentiation, and activation should be considered (12, 21, 38, 354, 355). Epigenetic, reversible regulation of Tregs toward higher ectopic endometrial cell detectability, and elimination can be a significant step in human treatment. Further studies are needed to investigate how such therapy influences coexisting autoimmune diseases (36, 356).

## Author contributions

As the only author of this review paper, I acknowledge my full contribution and responsibility for the content of the manuscript.

## Conflict of interest

The author declares that the research was conducted in the absence of any commercial or financial relationships that could be construed as a potential conflict of interest.​​​​​​​​​​​​​​​​​​​​

The handling editor APG declared a past co-authorship with the author.

## Publisher’s note

All claims expressed in this article are solely those of the authors and do not necessarily represent those of their affiliated organizations, or those of the publisher, the editors and the reviewers. Any product that may be evaluated in this article, or claim that may be made by its manufacturer, is not guaranteed or endorsed by the publisher.
